# The impact of climate change on medicinal plants and natural products: A scoping review

**DOI:** 10.3389/fphar.2025.1697581

**Published:** 2025-11-24

**Authors:** Marce Inggritha Takubessi, Banaz Jalil, Michael Heinrich

**Affiliations:** 1 Research Group “Pharmacognosy and Phytotherapy”, UCL School of Pharmacy, London, United Kingdom; 2 Pharmacy Department, Health Polytechnic of the Ministry of Health Kupang, Kupang, Indonesia; 3 Chinese Medicine Research Centre, College of Chinese Medicine, China Medical University, Taichung, Taiwan

**Keywords:** climate change, medicinal plants, bioactive natural products, traditional medicine, species distribution modeling, MaxEnt, GIS-based ecological assessments

## Abstract

**Background:**

Medicinal plants and natural products are essential for healthcare systems globally, and, at the same time, they are a part of ecosystems and have major socioeconomic importance in many regions of the world. However, climate change has threatened their supply and sustainability. In this review, we map the current state of research on how climate change affects medicinal plants, focusing on ecological shifts, traditional uses, changes in bioactive metabolites, and adaptation strategies.

**Methods:**

This scoping review, which was conducted following the PRISMA-ScR guidelines, involved comprehensive searches in PubMed, Scopus, and Web of Science of studies published between 2004 and 2024. Data were extracted to summarize study characteristics, climate change factors, species distribution, bioactive metabolites and marker compounds variations, and healthcare implications.

**Results:**

A total of 219 studies were included, showing a significant increase in publication after 2021. Most studies were conducted in Asia, especially in China and India, whereas Europe, Africa, and South America remain underrepresented. The review covers 367 medicinal plant species, including high-altitude, climate-sensitive species such as *Nardostachys jatamansi* and *Paris polyphylla*. Of these, 40.6% are classified as threatened by the IUCN, and 59.4% remain unevaluated, which shows significant conservation gaps. Research methods have evolved from basic experiments to advanced computational approaches, notably species distribution modeling (SDM), with MaxEnt being the most widely used. Although climate change is projected to increase habitat suitability for 70 species, it has also led to a decline in suitable habitats for 106 species, range shifts in 94 species, and placed 33 species at the risk of extinction and habitat loss. The ecological changes also impact traditional accessibility and the reliability of medicinal plant-based therapies. Moreover, shifts in bioactive metabolite production, including both increases and decreases, are linked to several environmental factors, such as rising temperatures, elevated CO_2_, reduced precipitation, and drought stress.

**Conclusion:**

Climate change is reshaping the ecology and pharmacological value of medicinal plants. Although adaptation strategies exist, their implementation remains limited. An interdisciplinary, coordinated response is urgently needed to ensure sustainable production and use. This will also require a paradigm shift in all aspects of ethnopharmacological research and development.

## 1 Introduction

Medicinal plants are fundamental to human health and well-being because of their extensive contributions to healthcare, ecosystem sustainability, and socioeconomic development. Several medications originate from plant sources, such as Taxol from *Taxus brevifolia* Nutt. (Kwak et al., 1995), artemisinin from *Artemisia annua* L. (Chen et al., 2017), morphine from *Papaver somniferum* L. (Yuan et al., 2016), and colchicine from *Colchicum autumnale* L. (Lee, 1999). In addition to these well-studied examples, many other plant species remain underexplored, representing the potential for novel therapeutic agents (Balunas and Kinghorn, 2005).

Beyond their medical applications, medicinal plants play a vital ecological role. They support key ecosystem functions by sustaining pollinator populations (Morton and Rafferty, 2017), enhancing soil fertility (Dybzinski et al., 2008), and promoting biodiversity, thus collectively maintaining the integrity and resilience of natural habitats (Tomlinson and Akerele, 2015), including climatic stability and climate change mitigation. They are an essential element of local cultures and economies. These ecosystem services are essential for environmental stability and combating ecosystem degradation, forming an integral part of their roles in ecosystem services (Kala, 2015; Schippmann et al., 2002).

Medicinal plants form the cornerstone of many local and traditional healthcare systems, especially in indigenous and rural communities, where they often serve as the primary or sole source of healthcare and well-being (Heinrich et al., 1998; Upa et al., 2023; Ambu et al., 2020). Their use is typically rooted in generations of empirical knowledge transmitted orally and encompassing locally adapted preparation, dosage, and application (WHO traditional medicine strategy: 2014-2023, 2013) practices. For instance, snowdrop (*Galanthus woronowii* Losinsk. and G. spp) has been used traditionally in the Caucasus region of Eastern Europe to manage poliomyelitis, and its active component was later developed into a medication for this disease and subsequently into a treatment for Alzheimer’s disease (Heinrich and Teoh, 2004). At the same time, *Angelica sinensis* (Oliv.) Diels (Dang gui) is widely employed in traditional Chinese medicine (TCM) (Lin et al., 2017). In addition, the sustainable management and conservation of medicinal plant resources have profound socioeconomic implications. In many regions, local communities depend on these plants for traditional healthcare practices and as a vital source of income and economic development (Nainwal and Singh, 2020; Asigbaase et al., 2023).

Climate change alters the temperature patterns, precipitation levels, and soil conditions, directly impacting the growth and yield of medicinal plants (Moisiienko et al., 2019) and phytochemical composition (Mansinhos et al., 2024; Laftouhi et al., 2023). It induces habitat loss, which threatens the survival of wild medicinal plant species, many of which are already under pressure from overharvesting (Groner et al., 2022). These changes are part of broader ecosystem shifts impacting multiple environmental aspects, such as reducing the availability and accessibility of key medicinal compounds, which affects both traditional and modern healthcare systems that rely on plant-derived preparations. Changes in the composition of (bioactive) metabolites are likely. For instance, drought conditions may increase the production of phenolic compounds in *Olea europaea* L. (Mansour-Gueddes et al., 2020) and *Lallemantia royleana* Benth (Omidi et al., 2018). At the same time, decreasing the amount of rosmarinic acid and salvianic acid in *Mentha pulegium* L. reduces their medicinal benefits (Laftouhi et al., 2023).

Therefore, in this scoping review, we aim to understand and assess the current research landscape and the interface of climate change and medicinal plant/food supplement research, as well as to define core questions that need to be developed further, by answering the following questions:

1. What are the study trends, study regions, plant species, and research methods used over the past 2 decades on climate change impacts on medicinal plants and natural products?
2. How does climate change affect the geographic distribution of medicinal plants?
3. What are the impacts of climate change on the phytochemical composition of medicinal plants, for example, bioactive metabolites or marker compounds?
4. How does the decline of medicinal plants due to climate change impact healthcare systems, particularly in regions dependent on medicinal plant-derived therapies?
5. What adaptation and conservation strategies can be used to mitigate the impact of climate change on medicinal plants?
6. What knowledge gaps exist in the current research on climate change and medicinal, natural products, and plants?

Given these challenges, understanding and assessing the impact of climate change on medicinal plants is essential and highly significant for ethnopharmacology. The findings of this research aim to inform future scientific inquiries, which will guide conservation efforts and shape policies aimed at preserving the plant biodiversity and the cultural heritage embedded in traditional medicine, as well as the industrial applications of medicinal plants and bioactive natural products.

## 2 Methods

### 2.1 Protocol

**FIGURE 1:**
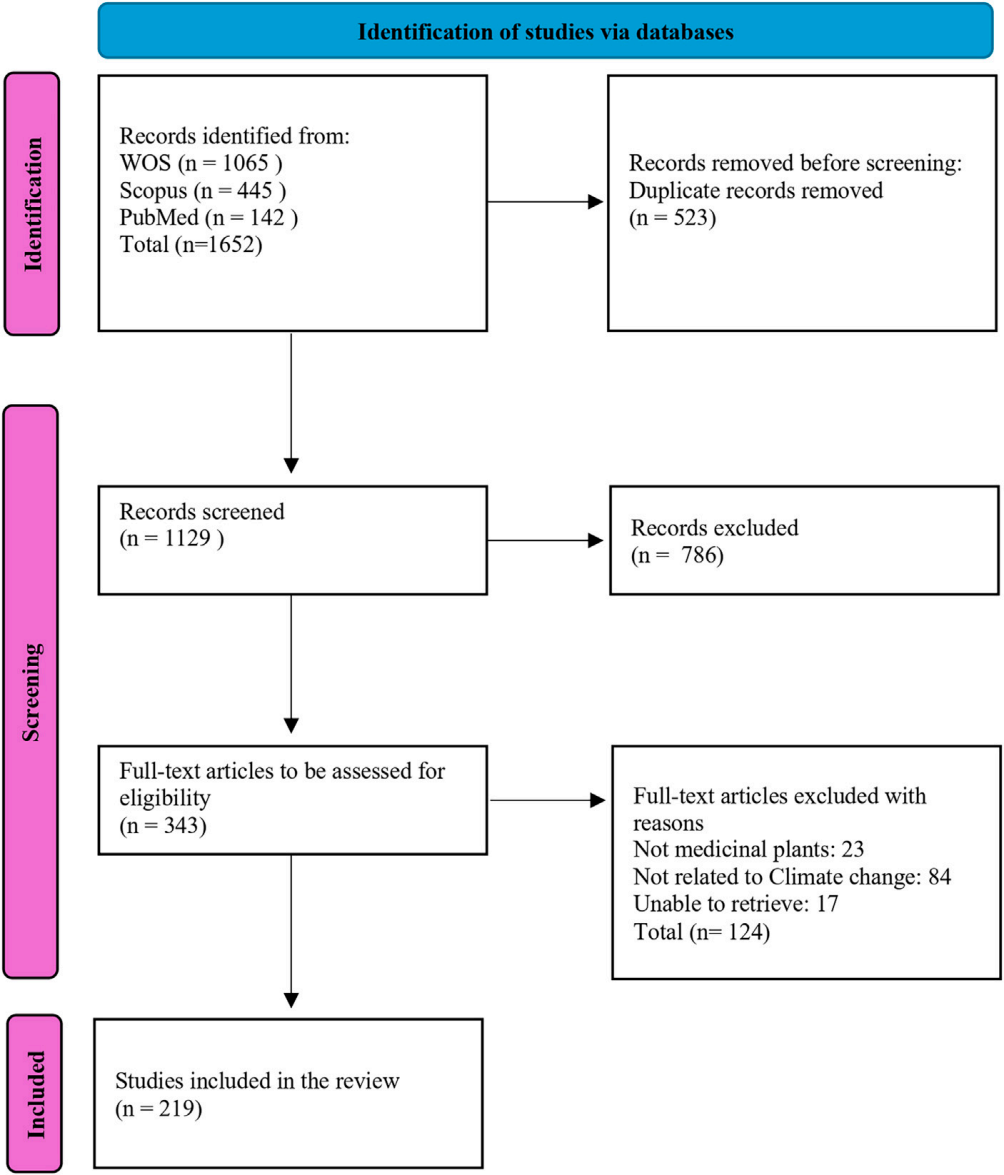
PRISMA flow diagram summarizing the search strategy, selection process, and the inclusion criteria (McGow et al., 2020).

The review followed the PRISMA-ScR (Preferred Reporting Items for Systematic Reviews and Meta-Analyses Extension for Scoping Reviews) guidelines (Tricco et al., 2018) (Figure 1).

### 2.2 Eligibility criteria

This scoping review follows a structured methodology to systematically explore the impact of climate change on the sustainability of medicinal plants. The eligibility criteria for study selection include peer-reviewed articles, reviews, and relevant gray literature, such as reports and theses that specifically investigate this relationship. Only studies published within the last 20 years (2004–2024) were considered to ensure relevance, capture recent trends, and inform future practices. Exclusion criteria include studies that do not directly address medicinal plants or climate change, articles unavailable in full text, and non-peer-reviewed opinion pieces unless they provide significant insights.

### 2.3 Type of sources

The information sources and search strategy involve comprehensive PubMed, Scopus, and Web of Science.

### 2.4 Search strategy

A comprehensive search for relevant sources was conducted using a structured combination of keywords to capture literature on the impact of climate change on medicinal plants. The search terms addressed key aspects, including species distribution, bioactive and marker compounds, and implications for healthcare systems. The following Boolean search string was applied across databases:

(“Climate Change” OR “Global Warming” OR “Climate Variation” OR “Environmental Change”) AND (“ Medicinal Plants” OR “Medicinal herbs” OR “Ethnobotany” OR ““Phytomedicine” OR “Natural Product”) AND (“ Species Distribution” OR Geographic Distribution” OR “Range shift OR “Habitat Change” OR “Ecological niche”) AND (“Secondary Metabolites” OR “Bioactive Compounds” OR “Phytochemical Composition” OR “Active Compound”) AND (“Healthcare” OR “Traditional Medicine” OR “Health System” OR “Medicinal Use” OR “Therapeutic Efficacy”) AND (“ Conservation” OR “Adaptation Strategies” OR “Sustainable Use” OR “Biodiversity Conservation”).

### 2.5 Selection of sources of evidence

The study selection process follows a two-stage approach: first, two independent reviewers will screen the titles and abstracts using Rayyan, with any disagreements resolved through discussion or by consulting a third reviewer. Second, a full-text review verifies the eligibility of selected studies based on the inclusion and exclusion criteria.

### 2.6 Data charting

Following PRISMA-ScR guidelines, a standardized data-charting form was jointly developed by two reviewers to determine the key variables for extraction. Both reviewers independently charted the data and subsequently met to compare results, resolve discrepancies, and refine the form. This iterative process ensured the consistency, transparency, and comprehensiveness of data extraction across all sources.

### 2.7 Data item

Data extraction is conducted using a standardized data charting form to collect key study details, including author(s), year of publication, study location, medicinal plant species studied, geographical origin, climate change factors (e.g., temperature increase and precipitation changes), and observed effects on plant growth, distribution, bioactive and marker compounds, and the healthcare system. Rayyan software was used to manage and organize data efficiently.

### 2.8 Synthesis of result

A descriptive summary is presented through a narrative synthesis, which facilitates data analysis and synthesis, highlighting key findings across various studies. Thematic analysis was also conducted to categorize information based on major topics such as changes in distribution shifts, habitats, alterations in secondary metabolite production, and the impact on the healthcare system. The results were visually represented through thematic maps, tables, and charts to enhance comprehension and support data interpretation (Figure 1).

## 3 Results and discussion

### 3.1 Study selection

In total, 1,652 records were identified, with 343 full-text articles assessed for eligibility. A total of 219 studies were included in this review (Figure 1).

### 3.2 Key findings

#### 3.2.1 Study trends

##### 3.2.1.1 Timeline and region concentration

**FIGURE 2:**
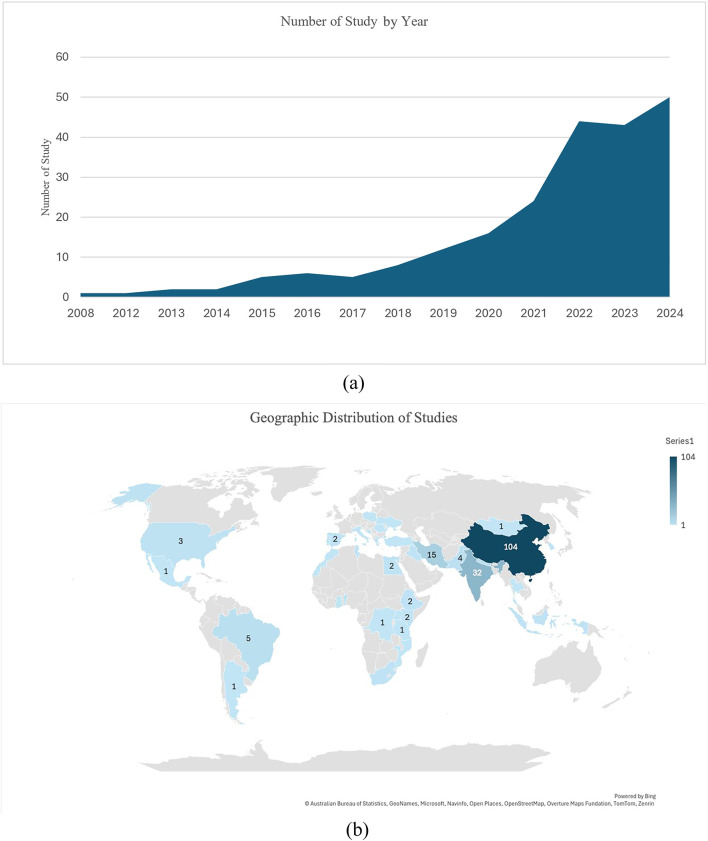
Studies trends by year **(a)** and region **(b)**.

The number of studies investigating the effects of climate change on medicinal plants has steadily increased over time. Between 2008 and 2016, research activity remained very low and gradual, with only 1–6 studies published annually. From 2017 to 2020, the number of studies began to rise steadily, reaching 16 studies in 2020. A notable surge occurred from 2021 onward, with research output nearly doubling compared to that of previous years. In both 2022 and 2023, over 40 studies were published each year. This upward trend peaked in 2024 with 50 studies, marking the highest annual research activity recorded (Figure 2).

The growing trend in publications between 2017 and 2020 suggests that scientists are becoming increasingly aware of the impact of climate change on medicinal plants. The post-2020 rise, which so far peaked in 2024, suggests an increasing need to examine changes in plant distribution, variety, and bioactivity—factors that are essential for the stability of ecosystems and conventional healthcare systems. This trend emphasizes how crucial the field is in directing conservation and sustainable resource management plans.

A total of 219 studies were included in this scoping review, revealing significant regional disparities in the distribution of research. The East Asia and Pacific region dominated, with 111 studies, overwhelmingly led by China (104), followed by very few from Indonesia (2), South Korea (2), Mongolia (1), Thailand (1), and general Asia (1). South Asia contributed 40 studies, primarily from India (32), with additional studies from Nepal (4) and Pakistan (4). The Middle East and North Africa (MENA) region accounted for 22 studies, with Iran (15) leading, and smaller contributions from Egypt (2), Iraq (2), Tunisia (2), and Morocco (1). Europe contributed 12 studies, led by Greece (3) and Spain (2), with single studies from Bosnia, Italy, Portugal, Romania, Turkey, Poland, and Ukraine.

Sub-Saharan Africa 15 studies come from Benin (3), South Africa (2), Kenya (2), Ethiopia (2), and single studies from Congo, Ghana, Mozambique, Tanzania, Uganda, and a general “Africa” category. Latin America and the Caribbean were represented by only ten studies –Brazil (5), and single studies from Argentina and Mexico. North America contributed three studies, all from the United States. Additionally, nine studies were categorized as global or cross-regional, including one at the continental scale in Africa. Overall, research is heavily concentrated in China and India, with limited representation from Sub-Saharan Africa, Latin America, North America, and Europe (Figure 2).

China’s firm cultural and medical reliance on medicinal plants, as well as the industry’s substantial economic importance (Wang W-Y. et al., 2021), underscores the need for proactive research on the effects of climate change. The natural habitats of high-value medicinal plants (e.g., *Fritillaria cirrhosa* D. Don) are becoming increasingly threatened by climate change, especially in areas with high biodiversity, such as the Tibetan Plateau (Cunningham et al., 2018) (Zhao et al., 2018). Another key driver behind the growing research on the effects of climate change on medicinal plants in China is the government’s recent push to promote the use of *Daodi* Chinese medicinal materials (CMMs), referring to region-specific medicinal materials traditionally recognized for superior quality and therapeutic efficacy. Therefore, specific measures are taken to understand the environmental conditions relevant for the *Daodi* status and more generally to ensure the quality of TCMs. Although these efforts aim to standardize and enhance the credibility of TCMs, the limited supply of CMMs from designated *Daodi* regions cannot meet the rising market demand. As a result, research has intensified to identify new or shifting suitable areas for cultivating *Daodi*-quality materials in response to changing environmental conditions (Zhao et al., 2012; Li et al., 2024).

##### 3.2.1.2 Methodologies used in the studies reviewed

**FIGURE 3:**
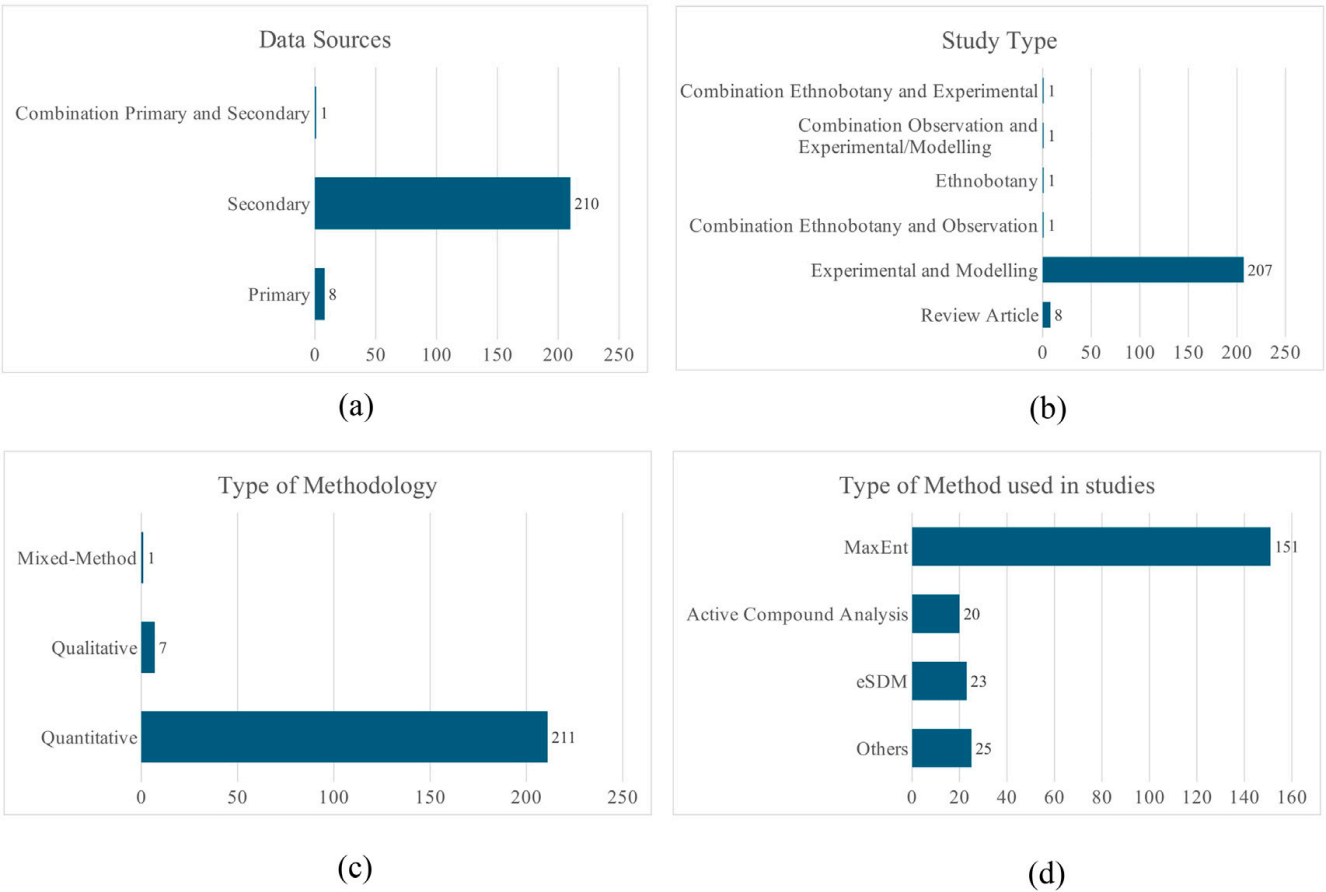
Methodology used in the studies reviewed: **(a)** data source, **(b)** study type, **(c)** type of methodology, and **(d)** type of method used in studies.

The data reveal a clear dominance of quantitative studies (211) compared to only seven qualitative and one mixed-method study. Experimental and modeling approaches account for 207 publications, whereas ethnobotanical and observational studies are exceptionally rare, with only a single instance each. Most of the research (210) is primary, whereas secondary studies number just eight, and only one study combines both approaches (Figure 3).

Study methods have evolved. For example, from 2000 to 2010, studies were limited and primarily relied on basic experimental methods, such as Open-Top Chambers (OTCs) and simulated grazing, to observe plant responses. Between 2011 and 2020, research activity increased, with 50 studies recorded—34 of which Species Distribution Modeling (SDM). Active compound analysis, genetic analysis, GIS-based ecological assessments, and integrated field surveys gained prominence during this period. The most significant growth occurred between 2021 and 2024, with 160 studies, including 125 using SDM. This phase marked a surge in interdisciplinary methods, combining SDM with biochemical, genetic, and molecular approaches such as transcriptomics and GIS-based climate modeling. Regarding SDM techniques, MaxEnt emerged as the dominant tool, with usage rising from 28 studies (2011–2020) to 99 (2021–2024). Ensemble SDM (eSDM) also grew in use, increasing from three to 21 studies. New machine learning methods, such as random forest (RF) and RF on the GGE platform, appeared during the 2021–2024 period (Figure 3).

##### 3.2.1.3 Species included in the studies reviewed

###### 3.2.1.3.1 Taxonomic and habitat information of the plants studied

In this study, we examine the impact of climate change on 367 medicinal plant species (See Supplementary Material Table S1). *Nardostachys jatamansi*, *Paris polyphylla*, *Dendrobium nobile*, and *Ephedra sinica* are identified as species of high medicinal value and with restricted geographical distribution. These species are predominantly located in high-altitude areas such as the Himalayas, the Tibetan Plateau, and the mountainous regions of China, Nepal, and India.

Regions such as the Himalayan belt, the Qinghai–Tibet Plateau, and parts of Central Asia harbor a rich biodiversity of medicinal plants. These areas have been the focus of extensive studies due to their ecological importance and the role in traditional medicine systems. In addition, reports from Iran, Turkey, and North America highlight climate-related impacts on medicinal species such as *Thymus vulgaris*, *Matricaria chamomilla*, and *Panax quinquefolius*.

Regarding the IUCN conservation status, the largest majority, 216 species (59%), are “not listed,” highlighting a considerable gap in conservation assessments. Ninety-three species (25.3%) are classified as least concern (LC), indicating that they are not currently at risk. However, 54 species (14.7%) fall into threatened categories, including critically endangered (CR: 9), endangered (EN: 21), vulnerable (VU: 19), and near threatened (NT: 5), emphasizing their heightened vulnerability to environmental change. Additionally, four species (1.1%) are listed as data deficient (DD) (See Supplementary Material Table 1).

High-value plant species, such as *N. jatamansi*, *Aquilaria malaccensis*, *Santalum album*, and *Angelica glauca*, which are classified as critically endangered, endangered, and vulnerable, play vital roles in the pharmaceutical and cosmetic industries and support traditional livelihoods and cultural practices. *Nardostachys jatamansi*, native to the Himalayas, is valued for its rhizomes, which contain jatamansone, a key compound used in both Ayurvedic and modern medicine for treating neurological and inflammatory disorders (Singh et al., 2013; Arora and Arora, 2016). Its essential oil is also used in perfumes and skincare for its calming effects (Bhagwan et al., 2024). *Aquilaria malaccensis*, the source of agarwood, produces resin with antimicrobial and sedative properties and is widely used in traditional medicine and high-end perfumery (Hashim et al., 2016). Its role in religious rituals and its economic importance to rural Southeast Asian communities are particularly threatened by overharvesting and changing rainfall patterns (Mykhailenko et al., 2025).

The habitat of *S. album*, or Indian sandalwood, another highly valued species, is threatened by climate change (Indrioko and Ratnaningrum, 2015; Ratnaningrum and Indrioko, 2015). Known for its fragrant oil rich in α- and β-santalol, it is used in antiseptics, anti-inflammatory treatments, and luxury cosmetics (Kumar et al., 2015; Saneja et al., 2009), providing another example. Similarly, *A. glauca*, a Himalayan herb, is valued for its aromatic roots and essential oils, which are traditionally used in treating respiratory, digestive, and menstrual ailments. The soothing and anti-inflammatory properties of its essential oil also make it a sought-after component in natural skincare and aromatherapy formulations (Kumar et al., 2022; Khaleeq-Uz-Zaman and Shafi, 2018). However, all these species face growing threats from climate-induced habitat loss, shifting phenology, and reduced production of bioactive compounds. This affects the pharmaceutical and cosmetic industries through unstable supply chains and altered product quality, and it threatens the cultural heritage and incomes of communities that depend on their sustainable use.

A significant conservation gap underscores the urgency for more comprehensive conservation assessments, particularly for neglected and understudied taxa. Several endemic medicinal plants, including *Nepeta crispa* (Mahmoodi et al., 2022), *Dicksonia blumei* (Cahyaningsih et al., 2021), *Sideritis* spp. (Munt et al., 2016), and *Gentiana quadrifaria* (Cahyaningsih et al., 2021), are at the risk of extinction but are not included on the IUCN Red List. This lack of acknowledgment restricts their access to financing, legal protection, and conservation attention. Overharvesting, habitat loss, and climate change threaten many of these species, yet they are often overlooked due to incomplete data or limited investigation. More inclusive and regionally informed assessments are needed to protect these resources, particularly those with ecological and medicinal significance. It also highlights that approximately 71% of the studied plant sources are wild-harvested, with 45% being classified as critically endangered, endangered, vulnerable, or near threatened of the total wild-harvested sources.

**FIGURE 4:**
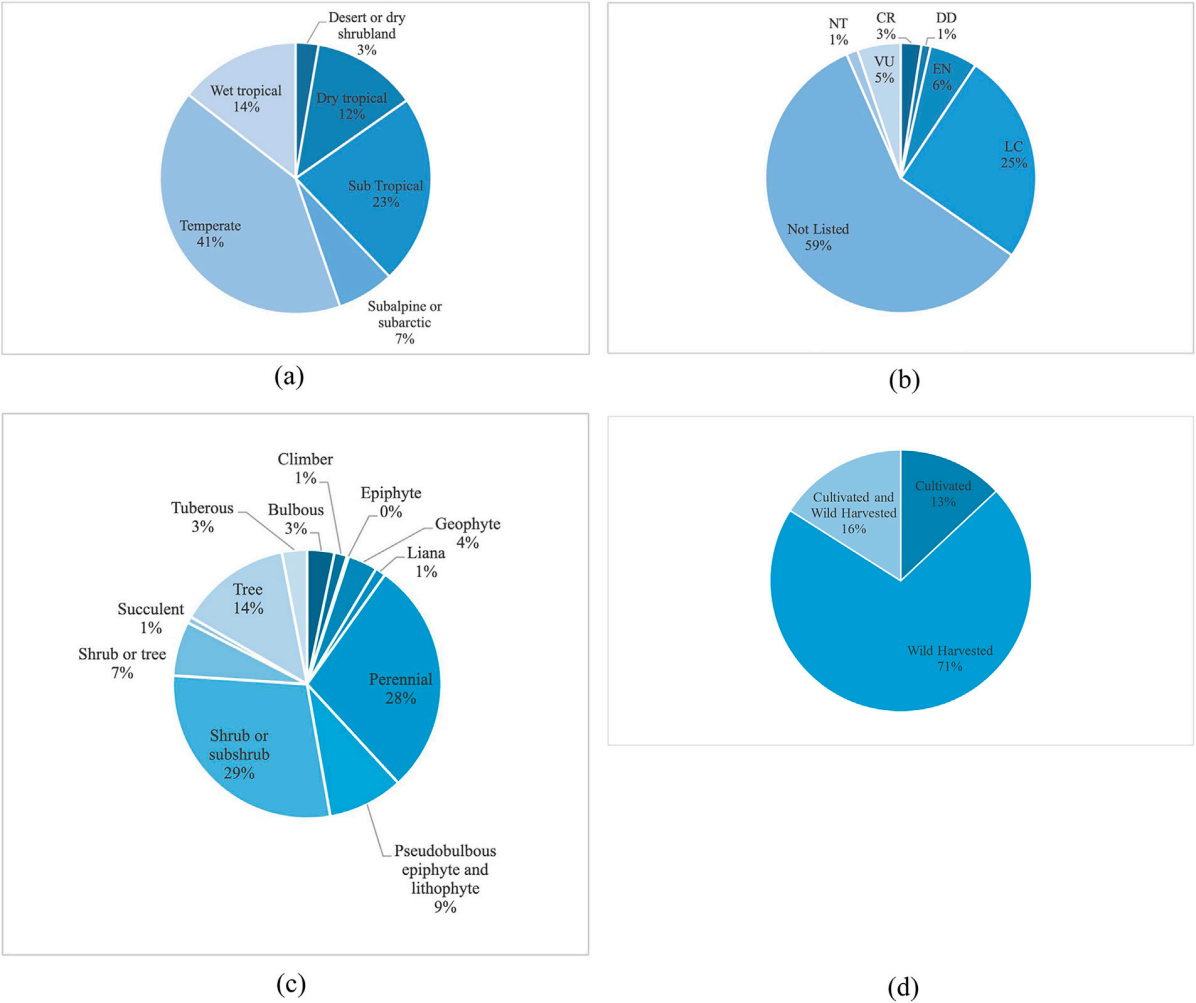
Studied plants. **(a)** Biome type of the plant. **(b)** IUCN status of the plant. **(c)** Type of plant. **(d)** Source of plant material.

The distribution of medicinal plant species across different biomes reveals that with 189 species, the temperate region is the most prominent habitat (51% of the total). This is followed by the subtropical biome, which includes 80 species (24.6%), indicating its vital role in sustaining medicinal plant diversity. Wet tropical regions also contribute significantly, hosting 51 species (15.2%), but with this being a highly species-rich biome, the value is relatively low. In contrast, dry tropical zones support 38 species (10.6%), whereas subalpine or subarctic areas contain 29 species (8.2%), reflecting a more moderate presence. The most underrepresented environments include desert or dry shrubland, with just four species (1.1%), and seasonally dry tropical biomes, with only one species (Figure 4).

##### 3.2.1.4 Habitat and species distribution

**FIGURE 5:**
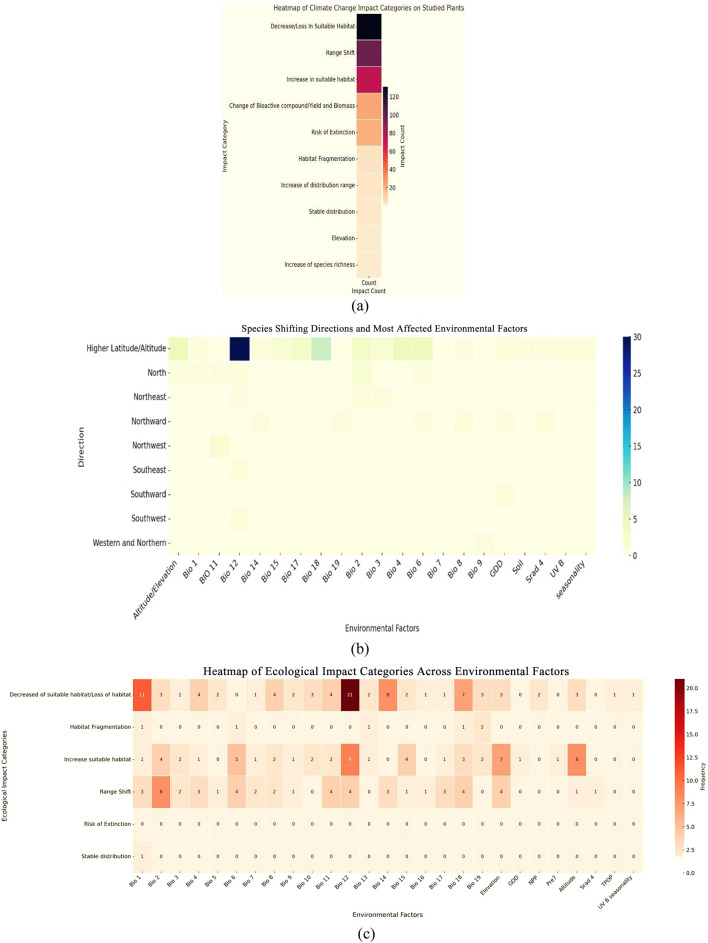
Prediction of the impact and environmental factors under future climate scenarios. **(a)** Type of impact. **(b)** Direction of shifting and contributing environmental factors. **(c)** Environmental factors that contribute to the impact.

The analysis reveals that the most frequent impact of climate change and specific aspects resulting from it on the plant species studied is the decrease or loss of suitable habitats, affecting 131 species. This is followed by a range shift (97 species) and an increase in suitable habitats (73 species). Changes in the bioactive metabolites, yield, or biomass were recorded in 24 species, whereas the risk of extinction was noted in 21 species. Other observed impacts include habitat fragmentation (four species), an increase in the distribution range (three species), stable distribution (two species), and isolated cases of species richness increase and elevation-specific responses (one species each). The loss of suitable habitat is the most widespread impact, influenced by over 20 variables, especially BIO 12 (annual precipitation) and elevation. An increase in appropriate habitat and range shift also strongly correlates with elevation and multiple bioclimatic factors. The heatmap indicates that plants shifting to higher latitudes and altitudes are associated with the broadest range of environmental factors, notably, Bio 12 and Bio 18 (precipitation of the warmest quarter). North and northward directions are associated with fewer but key factors, such as Bio 1 (annual mean temperature), Bio 2 (diurnal range), and growing degree days (GDD)—an index of accumulated thermal units above a base temperature, used to estimate biological development (Figure 5).

Climate change is predicted under future scenarios to significantly reshape the distribution and habitat of important medicinal plants, with many species projected to lose suitable environments or shift to higher altitudes and latitudes (Kumar et al., 2019; Hao et al., 2024; Souther and McGraw, 2014; Rajpoot et al., 2020). Whereas a few may expand their range (Liu et al., 2023; Bariotakis et al., 2023; Qiu et al., 2024), most face increased extinction risk (Souther and McGraw, 2014; Qiu et al., 2024; Wang W. et al., 2021) due to limited adaptability, habitat fragmentation, and climate extremes, particularly temperature, precipitation, and elevation changes. Mountain regions are predicted to be key transition zones under future climate scenarios (Pan et al., 2022; Jung et al., 2023; Singh et al., 2022). Non-climatic factors such as land use (Vihotogbé et al., 2021; Zhang et al., 2024) and overharvesting (Groner et al., 2022; Ngaruiya, 2015) also contribute to species vulnerability.

Shifting of species distribution implies a change in the availability of medicinal plants in the pharmaceutical and cosmetic industries. This change potentially disrupts the pharmaceutical value chains (Mykhailenko et al., 2025; Lv et al., 2024; Singh et al., 2024). Pharmaceutically, the production of herbal medicinal products depends on cultivated and wild-harvested medicinal plants, making them highly vulnerable to climate-induced changes in plant growth, distribution, and productivity. As temperature and precipitation patterns shift, previously viable growing areas may become unsuitable under specific climate scenarios (Ngarega et al., 2024), which can reduce yields (Omidi et al., 2018) and disrupt the distribution of the starting plant materials. Moreover, the risk of extinction of some medicinal plants leads to a loss of opportunity to explore new therapeutic agents (Brower, 2008; Mendelsohn and Balick, 1995; Newman et al., 2003).

Climate change-induced shifts in the species distribution may profoundly impact geographical indications (GIs), which are designations for products whose unique qualities, reputation, or characteristics are inherently linked to the geographic origin of the medicinal plants, including traditional concepts such as *Daodi* and European-recognized notions of provenance and terroir. These terms represent the unique qualities attributed to specific regions, which are shaped by their environmental conditions, cultivation practices, and cultural heritage. However, as temperature, precipitation patterns, and atmospheric CO_2_ levels change, the optimal habitats for many medicinal plants are shifting or contracting. This disrupts the ecological foundations that give these regions their distinctive phytochemical profiles.

Consequently, zones designated for geographical indications, such as *Daodi* regions or established terroirs, may need to shift in response to changing environmental conditions. As optimal growing regions are redefined, this could lead to the relocation or reclassification of GI zones. Such transitions pose significant challenges to preserving traditional quality standards and underscore the need for adaptive frameworks capable of reassessing and updating GI designations in response to ecological change (Brinckmann, , 2015).

##### 3.2.1.5 Bioactive metabolites and marker compounds

Global warming may significantly alter the production of bioactive metabolites and marker compounds in medicinal and aromatic plants by increasing potential evapotranspiration due to elevated temperatures, which in turn accelerates soil drying (Trenberth et al., 2014). The resulting changes in the phytochemical profiles are highly variable and depend on the plant species, the specific type of compounds, and the intensity and duration of drought stress (Tan and Gören, 2024). Moderate drought often enhances the accumulation of various secondary metabolites, especially those with antioxidant properties. This is attributed to the induction of mild oxidative stress, which triggers the biosynthesis of protective compounds such as phenolics and flavonoids to scavenge reactive oxygen species (ROS) (Ai et al., 2024). When drought becomes prolonged and severe, plant metabolic processes are significantly disrupted (Li et al., 2020). This contrasting pattern was evident in several species that were reviewed. For example, under water-deficit conditions, both *Lallemantia royleana* and *Melia azedarach* exhibited increased phenolic and flavonoid contents (Omidi et al., 2018; Dias et al., 2022). However, in *Lavandula latifolia*, drought conditions resulted in decreased levels of specific metabolites such as coumaric and salvianic acids (Cáceres-Cevallos et al., 2023) (Table 1).

**TABLE 1:** Impact of environmental factors on specific bioactive metabolites and marker compounds.

Environmental factor	Compound	Impact	Species
Drought stress/water deficit	Phenolic compounds	Increased	*Lallemantia royleana* (Omidi et al., 2018)
			*Melia azedarach* (Dias et al., 2022)
			*Olea europaea* (Mansour-Gueddes et al., 2020)
			*Lavandula latifolia* (Cáceres-Cevallos et al., 2023)
	p-Coumaric acid glycoside	Decreased	*Lavandula latifolia* (Cáceres-Cevallos et al., 2023)
	Rosmarinic acid-3–O-glucoside	Increased	*Lavandula latifolia* (Cáceres-Cevallos et al., 2023)
	Salvianic acid	Decreased	*Lavandula latifolia* (Cáceres-Cevallos et al., 2023)
	O-Coumaric acid	Decreased	*Lavandula latifolia* (Cáceres-Cevallos et al., 2023)
	Flavonoid	Increased	*Melia azedarach* (Dias et al., 2022)
	α-Linolenic acid	Increased	*Melia azedarach* (Dias et al., 2022)
Elevated CO_2_ and temperature	Phenolic compounds	Increased	*Angelica glauca* (Dobhal et al., 2024)
		Decreased	*Nardostachys jatamansi* (Dobhal et al., 2024)
			*Gynostemma pentaphyllum* (Chang et al., 2016)
Elevated temperature	Coumarins, flavonoids, tannins, and alkaloids	Decreased	*Mentha pulegium* (Laftouhi et al., 2023)
	Tannins	Decreased	*Mentha pulegium* (Laftouhi et al., 2023)
			*Populus tremuloides* (Netshiluvhi and Eloff, 2024)
	Alkaloid	Decreased	*Mentha pulegium* (Laftouhi et al., 2023)
	Phenols	Decreased	*Myrtus communis* (Medda et al., 2022)
High altitude/latitude	Phenolic compounds	Increased	*Aloe vera* (Kumar et al., 2017)
			*Hypericum perforatum* (Kaur et al., 2016)
	Gentiopicroside	Increased	*Gentiana rigescens* (Shen et al., 2021)
	Flavonoids	Increased	*Hypericum perforatum* (Kaur et al., 2016)
			*Matricaria chamomilla* (Kaur et al., 2016)
			*Thymus vulgaris* (Kaur et al., 2016)
			*Cynara cardunculus* (Kaur et al., 2016)
			*Echinacea purpurea* (Kaur et al., 2016)
	Anthocyanins	Increased	*Myrtus communis* (Medda et al., 2022)
Increasing of precipitation	Gentiopicroside	Increased	*Gentiana rigescens* (Shen et al., 2021)
Reducing of precipitation	Coumarins, flavonoids, tannins, and alkaloids	Decreased	*Mentha pulegium* (Laftouhi et al., 2023)
Temperature stress	VOCs	Increased	*Ocimum basilicum* (Copolovici et al., 2022)

As a major contributor to climate change, elevated CO_2_ and temperature exhibit mixed results; in certain cases, they can enhance the production of some compounds while suppressing others due to complex carbon allocation and temperature sensitivities (Jamloki et al., 2021). High temperatures alone can reduce the levels of several secondary metabolites, which is likely due to metabolic shifts that favor survival over defense (Li, 2020).

In contrast, high-altitude and latitude environments generally promote the synthesis of phenolics, flavonoids, and anthocyanins, which is possibly due to increased UV exposure and cooler temperatures that trigger protective pathways (Ashraf et al., 2024). Changes in precipitation also influence metabolite levels, with reduced rainfall often linked to a decline in compound diversity, particularly in drought-sensitive species (Prinsloo and Nogemane, 2018). In addition, temperature stress elevates volatile organic compounds, suggesting an upregulation of stress signaling or defense responses (Midzi et al., 2022) (Table 1).

These fluctuations in secondary bioactive metabolite and marker compound profiles could impact the potential risks and benefits of medicinal plants. Increased levels of certain compounds could enhance the antioxidant, anti-inflammatory, or antimicrobial activity, whereas a decline in key bio-actives may compromise the potential effectiveness of traditional and plant-derived preparations (Kumar et al., 2017; Kaur et al., 2016).

Similarly, the pharmaceutical and cosmetic industries face significant challenges. Drought, for instance, alters the synthesis of essential oils, phenolic compounds, and flavonoids, which are key compounds in skincare, personal, and healthcare products. Changes in the levels of bioactive metabolites or marker compounds—such as linalool, linalyl acetate, α-pinene, and borneol in lavender (Jalil and Heinrich, 2025); camphor and linalool in basil; and various compounds in rosemary—due to drought and/or heat stress could explain these biochemical shifts (Kulak, 2020; Sarmoum et al., 2019).

These phytochemical shifts (including changes in the levels of bioactive metabolites or marker compounds) can affect the quality (including the safety and efficacy of plant-derived medical products) and sensory properties of cosmetic and healthcare products (Jalil and Heinrich, 2025) (Table 1).

However, much detailed information is needed to understand the impact of climate change, and no *a priori* predictions are plausible. It is an area where longer term and comparative studies are needed.

##### 3.2.1.6 Traditional healthcare

Climate change poses a serious threat to biodiversity and traditional healthcare systems that rely on medicinal plants. In the Central Himalayas of India, rising temperatures and shifting precipitation patterns have advanced the flowering and fruiting times of key species by 15–30 days, disrupting traditional harvesting practices rooted in cultural and spiritual traditions. Research identified 15 high-value medicinal plants commonly used by traditional healers, many of which are declining in availability and effectiveness due to phenological shifts. As a result, healers are increasingly turning to substitute species, which are reported to be 10%–40% less effective (Maikhuri et al., 2018). Similarly, in northern Thailand, eight out of nine key medicinal species used by Karen women are projected to lose significant suitable habitat by 2050 and 2080, with six potentially becoming critically endangered. These changes directly threaten local healthcare practices, especially those related to women’s reproductive health, and highlight the urgent need for conservation and climate adaptation strategies. Only one species is expected to gain a suitable area. These changes threaten the availability of medicinal plants, particularly affecting the reproductive health of Karen women (Tangjitman et al., 2015).

Similar challenges are faced by the Shan people of Myanmar and forest-dependent communities near the Chunati Wildlife Sanctuary in southeastern Bangladesh. These populations, already affected by prolonged social instability and inadequate public healthcare, rely extensively on traditional primary healthcare systems for their medical needs (Mon et al., 2024; Rahman et al., 2022). As climate change alters the distribution and potency of medicinal plants, their ability to sustain these systems is increasingly under threat.

## 4 General discussion

In this study, we provide evidence on the impacts of climate change on medicinal plants and natural products, with significant implications for ethnopharmacology, cultural heritage, socioeconomic values, pharmaceutical development and industry, livelihoods, and biodiversity conservation. From an ethnopharmacological perspective, climate-induced shifts in plant distribution, phenology, and bioactive metabolites and marker compounds could impact and disrupt traditional healthcare systems that rely on specific medicinal species. Communities in regions such as the Central Himalayas and Northern Thailand are witnessing a decline in the availability and effectiveness of key medicinal plants, leading to increased use of substitute species that are often less effective (Maikhuri et al., 2018; Tangjitman et al., 2015) or less well understood in terms of potential risks. This undermines the potential benefits of traditional healing practices and poses a risk to these communities.

Culturally, medicinal plants hold deep spiritual and heritage value, often tied to specific harvesting times and rituals. Climate-driven changes threaten these cultural practices by altering the timing and availability of culturally significant species, thus potentially eroding traditions passed down through generation (Cunningham, 2014).

In economic terms, millions of people, particularly in rural and forest-dependent communities, are closely tied to the harvesting and selling of medicinal plants. As suitable habitats shrink and yields decline, these populations face increasing economic vulnerability (Palit et al., 2021). Promoting sustainable, climate-resilient cultivation practices and supporting local value chains can help safeguard these livelihoods while reducing pressure on wild plant populations (Fajinmi et al., 2023).

Biodiversity and ecosystem health are also at risk as many medicinal plants, particularly those endemic to high-altitude or climate-sensitive areas, face extinction or habitat loss. The ecological functions these plants perform, from soil stabilization to supporting pollinators, mean that their loss could destabilize entire ecosystems. Conservation efforts must, therefore, be expanded to include high-profile species and underrepresented and culturally important taxa. Integrated conservation strategies are vital, combining germplasm preservation, *in situ* and *ex situ* methods, and community-based stewardship.

From a pharmaceutical perspective, the results indicate that climate change alters the phytochemical composition of medicinal plants, potentially impacting the safety, efficacy, and dosage of herbal medicines and their derived pharmaceutical products. For instance, elevated temperatures and CO_2_ levels may increase the biomass but reduce the concentrations of specific compounds, such as flavonoids and phenolics, thereby compromising medicinal quality (Chang et al., 2016). Here, further research will be needed to assess differences in the responses to climate change as it relates to the composition of metabolites.

In the cosmetics industry, environmental factors, such as droughts driven by climate change, also alter the biochemical composition of plant species that are widely used in cosmetics. In addition, water stress can significantly influence the synthesis of bioactive metabolites and marker compounds such as essential oils, antioxidants, and flavonoids, which are key ingredients in skincare and personal-care products. For instance, α-pinene, D-limonene, eucalyptol, and *Champora* in rosemary, lavender, basil, and sage show significant variation under water-limited conditions (Kulak, 2020). These biochemical shifts can affect the quality, efficacy, and sensory properties of cosmetic and personal-care products, posing a challenge for standardization and consistency in plant-based cosmetics. Consequently, this underscores the importance of understanding plant responses to climate-induced stress and developing resilient cultivation practices.

Overall, in this study, we emphasize that climate change affects medicinal plants and natural products and, therefore, ethnopharmacology as a field of research.

Research gaps and recommendations: in this scoping review, we have identified a series of core research gaps:

- Geographic bias: research is heavily skewed toward regions with strong scientific infrastructure, leaving biodiversity hotspots, such as Southeast Asia, sub-Saharan Africa, Central America, and the Amazon, critically underrepresented. These areas are rich in both medicinal plant diversity and traditional knowledge, yet they remain marginalized in global research efforts.
- Similarly, some core areas of consumption, such as North America and Europe, are very poorly represented.
- Study design limitations: most existing studies are short-term, laboratory-based, or reliant on ecological modeling. This approach offers limited real-world applicability and fails to capture dynamic, long-term ecological and cultural changes. There is a pressing need for interdisciplinary, field-based research that integrates ethnobotany, climate science, and ecology.
- Lack of a standardized assessment framework: there is no unified model or standardized framework for assessing climate vulnerability and resilience along the medicinal plant supply chain. This makes it difficult to compare findings across regions and species or to prioritize conservation actions systematically.
- Neglect of biotic and human influences: current research often overlooks key ecological interactions, such as pollination networks, invasive species, competition, and human drivers such as overharvesting, land-use change, and cultural practices. These factors play a crucial role in shaping the survival of medicinal plants and ecosystem dynamics under climate stress.
- The high proportion of unassessed species or outdated assessments: a substantial number of medicinal plant species have yet to be evaluated for their conservation status or climate vulnerability. This gap highlights the urgent need for comprehensive biodiversity assessments to guide targeted conservation strategies before species decline becomes irreversible.
- Sociocultural and economic dimensions are often overlooked: traditional knowledge systems, cultural practices, and the bioeconomic value of medicinal plants are frequently inadequately addressed. These sociocultural aspects are crucial for preserving indigenous healthcare systems and guiding sustainable management and policy frameworks that support local communities.

## 5 Implications for public policy and conservation practices

The synthesis of findings from this review underscores several cross-cutting themes that can guide future conservation and policy action. These themes emphasize the integration of ecological resilience, inclusive governance, and socioeconomic sustainability to ensure long-term biodiversity and ecosystem security.

- Integrating Climate-Resilient Land Management and Agroforestry

Agroforestry and climate-resilient practices offer pathways to reduce pressure on wild populations while improving ecological stability. Although promising, both actions in this category rely on a single reference (Giliba and Yengoh, 2020; Groner et al., 2022; Feng et al., 2020), suggesting that the field is still in its early stages of development. Broader empirical support and implementation frameworks are needed to guide the integration of this approach into mainstream conservation and land‐use planning.

- Advancing Species Management and Genetic Conservation

The conservation of genetic diversity remains a cornerstone of long-term resilience. Actions such as germplasm collection, tissue culture, and gene banking (Zhang et al., 2024; Shen et al., 2021; Peralta et al., 2024; Zou et al., 2024) ensure preservation in both contracting and climatically stable areas. However, while most strategies are well-supported, the lack of a cited reference for socio-economic balancing in germplasm management highlights a gap in the literature. Future work should better integrate social sustainability within technical conservation frameworks.

- Mainstreaming Inclusive and Participatory Conservation

Recognition of Traditional Ecological Knowledge (TEK) and community engagement has gained momentum. These strategies emphasise the importance of participatory conservation, sustainable harvesting, and co-development of management plans. This evidence supports the shift toward more inclusive, culturally embedded conservation models, with community stewardship at the core (Maikhuri et al., 2018). Conservation practice must transition from top-down implementation to co-developed, place-based management that aligns scientific and cultural perspectives.

- Strengthening policy coherence, regulation, and incentives

Policies that strengthen biodiversity protection, reduce illegal harvesting, and promote incentives like Payments for Ecosystem Services (PES) are well-represented (Zou et al., 2024). This reflects growing alignment between environmental legislation and conservation priorities. The documented inclusion of international agreements and governance frameworks shows a maturing policy environment that supports cross-sector conservation integration.

- Training and research needs

This scoping review has important implications for developing research strategies in this field. It points to the need to systematically develop assessments of the climate change’s impact in some of the main regions, where medicinal plants are produced, including, for example, the MENA (Middle East and North Africa) region, South America, Southern Africa, large parts of Europe, and Southern/South-Eastern Asia. These knowledge gaps have major implications for species protection, local development, international trade and incorporating such resources into measures for environmental protection. Obviously, funding schemes are needed, which enable such research and the relevant training of postgraduate researchers and students.

## 6 Conclusion

Over the next 2 decades, climate change is expected to affect the distribution, accessibility, availability, and optimal growth conditions of medicinal plants, as well as other high-value plants used for their bioactive metabolites. Environmental factors may also impact and alter the bioactive metabolites and marker compounds, reducing the efficacy, safety, and consistency of plant-derived pharmaceutical, cosmetic, and food supplement preparations. As a result, communities that rely on these plants for their livelihoods, cultural heritage, and economic value, along with both contemporary and traditional healthcare systems, could become increasingly vulnerable.

Disruptions in the supply chain of medicinal plants could significantly impact several industries, including the pharmaceutical and cosmetic industries that rely on the sustainable, consistent, and resilient sourcing and supply of medicinal plants and natural products, which may also restrict access to essential natural products if conservation efforts and research are not effectively coordinated. This is also relevant as some regulatory systems require specific certifications of source regions, and if these shift, regulatory changes will be essential.

In the era of changing climate, relevant industries need to identify and understand how climate change affects secondary bioactive metabolite and marker compounds, which can aid in prioritizing specific species for further research, improving sourcing strategies, or anticipating variability in plant-derived raw materials (i.e., primary plant materials) and finished products.

In this work, we also provide a critical evidence base for integrating environmental factors into the drug development pipeline and align pharmaceutical goals with broader sustainability and conservation imperatives. As such, we address the foundational requirements in the field of ethnopharmacology/medicinal plant and natural product research.

This review calls for an integrated and multidisciplinary strategy that combines ecology, pharmacology, ethnobotany, and policy to address these challenges, is embedded in the framework of the United Nations Sustainable Development Goals (SDGs), and provides actionable and practical recommendations as needed, especially within the most vulnerable communities.

## 7 Limitation

This review has several limitations. The exclusive use of PubMed, Scopus, and Web of Science may have restricted regional and linguistic diversity, as these databases primarily index English-language and internationally oriented journals. Consequently, relevant studies published in regional or non-indexed journals may have been overlooked. “Gray literature” is relevant but difficult to capture. These limitations reflect both the methodological focus of scoping reviews, which aim to map the breadth and nature of peer-reviewed evidence rather than exhaustively synthesize all sources, and the limited availability and reliability of data within gray literature. The scarcity and inconsistency of such data further constrained its inclusion. Nonetheless, the scoping review approach remains appropriate for identifying the extent, range, and nature of existing research and for highlighting key gaps that require future investigation.

## Data Availability

The original contributions presented in the study are included in the article/Supplementary Material further inquiries can be directed to the corresponding author.
