# Supplementary material for: The impact of climate change on medicinal plants and natural products: A scoping review
Source: Front Pharmacol. 2025 Nov 24;16:1697581. doi: 10.3389/fphar.2025.1697581 (PMC12682780; doi:10.3389/fphar.2025.1697581)
Supplement: Supplementary file 1 [file Table1.pdf]

**Supplementary: Species identified and included in this scoping review (n=367)**										
**No**	**Species**	**Family**	**Accepted Species**	**Synonym**	**IUCN Status**	**Plant Type**	**Native Range**	**Climate type**	**Cultivated/ Wild Harvested**	**Refer ence**
1	*Acacia etbaica*	Fabaceae	*Acacia etbaica* Schweinf.	*Vachellia etbaica (Schweinf.) Kyal. & Boatwr.*	LC	Shrub or Tree	SE. Egypt to Tanzania, Arabian Peninsula	Desert or dry shrubland biome	Wild Harvested	(101)
2	*Acacia mellifera*	Fabaceae	*Acacia mellifera* (Vahl) Bosc.	*Senegalia mellifera (Vahl) Seigler & Ebinger*	LC	Shrub or Tree	Egypt to S. Africa, Arabian Peninsula	Desert or dry shrubland biome	Wild Harvested	(101)
3	*Acacia tortilis*		*Acacia tortilis (Forssk.)* Hayne.	*Vachellia tortilis (Forssk.) Galasso & Banfi*	LC	Shrub or Tree	Africa, Sinai to Arabian Peninsula	Desert or dry shrubland biome	Wild Harves ted	(101)
4	*Acacia xanthophloea*	Fabaceae	*Acacia xanthophloea* Benth.	*Vachellia xanthophlo ea* (Benth.) Banfi & Galasso	LC	Tree	N. Somalia to S. Africa	Dry tropical	Wild Harvested	(101)
5	*Achillea eriophora*	Asteraceae	*Achillea eriophora* DC.		Not Listed	Shrub	Iran	Dry tropical	Wild Harvested	(102)
6	*Aconitum brunneum*	Ranunculaceae	*Aconitum brunneum* Hand.-Mazz.		Not Listed	Tuberous geophyte	SE. Qinghai to China (NW. Sichuan, SW. Gansu)	Subalpine or subarctic	Wild Harvested	(103)
7	*Aconitum heterophyllum*	Ranunculaceae	*Aconitum heterophyllum* Wall. ex Royle		EN	Perennial or Tuberous Geophyte	N. Pakistan to Central Himalaya	Temperate	Wild Harvested	(104)
8	*Aconitum spicatum*	Ranunculaceae	*Aconitum spicatum* (Brühl) Stapf.	*Aconitum lethale* Griff.	Not Listed	Perennial or Tuberous Geophyte	Himalaya to S. Tibet.	Temperate	Wild Harvested	(105), (106)

9	*Agastache rugosa*	Lamiaceae	*Agastache rugosa* (Fisch. & C.A.Mey.) Kuntze		Not listed	Perennial	*Russian Far East to Temp. E. Asia.*	Temperate	Wild Harvested	(107)
10	*Agathis borneensis*	Araucariaceae	*Agathis dammara* (Lamb.) Poir.		EN	Tree	W. Malesia	Wet tropical	Wild Harvested	(44)
11	*Akebia quinata*	Lardizabalaceae	*Akebia quinata* (Thunb. ex Houtt.) Decne.		Not Listed	Liana	Central & E. China to Central & S. Japan	Temperate	Cultivated and Wild Harvested	(108)
12	*Akebia trifoliata*	Lardizabalaceae	*Akebia trifoliata* (Thunb.) Koidz.		Not Listed	Liana	Central & S. China to Japan.	Temperate	Wild Harvested	(109)
13	*Allium wallichii*	Amaryllidaceae	*Allium wallichii* Kunth		Not Listed	Bulbous geophyte	Pakistan to S. China and N. Indo-China	Temperate	Cultivated and Wild Harvested	(105)
14	*Aloe ferox*	Asphodelaceae	*Aloe ferox* Mill.		Not Listed	Succulent shrub or Tree	Cape Prov. to Lesotho	Subtropical	Wild Harvested	(110)
15	*Aloe vera*	Liliaceae	*Aloe vera* (L.) Burm.f.		Not Listed	Succulent Perennial	N. Oman (Hajar Mountains	Desert or dry shrubland	Cultivation	(73)
16	*Alstonia boonei*	Apocynaceae	*Alstonia boonei* De Wild.		LC	Shrub	W. Tropical Africa to Ethiopia and Tanzania	Temperate	Wild Harvested	(111)
17	*Alstonia iwahigensis*	Apocynaceae	*Alstonia iwahigensis* Elmer.		LC	Tree	Borneo to Philippines (Palawan)	Wet tropical	Wild Harvested	(44)
18	*Amburana cearensis*	Anacardiaceae	*Amburana cearensis* (Allemão) A.C.Sm.		EN	Tree	Bolivia to Brazil and NW. Argentina	Dry tropical	Wild Harvested	(112)
19	*Ammopiptanthus mongolicus*	Fabaceae	*Ammopiptanthus mongolicus* (Maxim. ex Kom.) S.H.Cheng.		Not Listed	Shrub	Mongolia to N. China.	Temperate	Wild Harvested	(113)
20	*Anadenanthera colubrina*	Cactaceae	*Anadenanthera colubrina* (Vell.) Brenan		LC	Shrub or Tree	Bolivia to Brazil	Dry tropical	Wild Harvested	(112)
21	*Anaxagorea javanica*	Annonaceae	*Anaxagorea javanica* Blume.		LC	Shrub or Tree	Andaman & Nicobar Islands	Wet tropical	Wild Harvested	(44)

							to W. & Central Malesia			
22	*Androsace elatior*	Primulaceae	*Androsace elatior* Pax & K.Hoffm.		Not Listed	Perennial	S. Qinghai to China (NW. Sichuan)	Subalpine or subarctic	Wild Harvested	(103)
23	*Angelica dahurica*	Apiaceae	*Angelica dahurica* (Hoffm.) Benth. & Hook.f. ex Franch. & Sav.		Not Listed	Perennial	*S. Siberia to N. China and Japan*	Temperate	Cultivated and Wild Harvested	(114)
24	*Angelica gigas*	Apiaceae	*Angelica gigas* Nakai.		Not Listed	Biennial or Perennial	*NE. China to Korea.*	Temperate	Cultivated and Wild Harvested	(55)
25	*Angelica glauca*	Apiaceae	*Angelica glauca* Edgew.		Not Listed	Perennial	E. Afghanistan to W. Tibet and W. Himalaya	Temperate	Wild Harvested	(69)
26	*Anisodus tanguticus*	Solanaceae	*Anisodus tanguticus* (Maxim.) Pascher.		Not Listed	Perennial	*Nepal to Central China*	Subalpine or Subarctic	Wild Harvested	(115)
27	Anisoptera costata	Dipterocarpaceae	*Anisoptera costata* Korth.		EN	Tree	Indo-China to W. & Central Malesia	Wet tropical	Wild Harvested	(44)
28	Aquilaria malaccensis	Thymelaeaceae	*Aquilaria malaccensis* Lam.		CR	Tree	Bangladesh to W. & Central Malesia	Dry tropical	Cultivated and Wild Harvested	(44)
29	*Aquilegia fragrans*	Ranunculaceae	*Aquilegia fragrans* Benth.		Not Listed	Perennial	*E. Afghanistan to W. Himalaya*	Temperate	Wild Harvested	(116)
30	*Arenaria brevipetala*	Caryophyllaceae	Arenaria brevipetala Tsui & L.H.Zhou	*Eremogone brevipetala* (Tsui & L.H.Zhou) Sadeghian & Zarre	Not Listed	Subshrub	NE. Tibet to SE. Qinghai and China (NW. Sichuan).	Subalpine or Subarctic	Wild Harvested	(103)
31	*Arisaema heterophyllum*	Araceae	*Arisaema heterophyllum* Blume		LC	Tuberous geophyte	*China to Temp. E. Asia*	Temperate	Wild Harvested	(117)
32	*Arisaema jacquemontii*	Araceae	Arisaema jacquemontii Blume		LC	Tuberous geophyte	Afghanistan to China (W. Yunnan), SW. India	Temperate	Wild Harvested	(118)
33	*Aristolochia indica*	Aristolochiaceae	Aristolochia indica L.		Not Listed	Climber	Indian Subcontinent to	Dry tropical	Wild Harvested	(119)

							Myanmar, Andaman Islands			
34	*Artemisia annua*	Asteraceae	*Artemisia annua* L.		Not Listed		*N. Africa to Eurasia*	Temperate	Cultivated and Wild Harvested	(120)
35	Artemisia granatensis	Asteraceae	*Artemisia granatensis* Boiss.		EN	Subshrub	S. Spain (Sierra Nevada)	Subalpine or Subarctic	Wild Harvested	(45)
36	*Asparagus cochinchinensis*	Asparagaceae	*Asparagus cochinchinensis* (Lour.) Merr.		DD	Perennial	*Japan to Indo-China and Philippines (N. Luzon)*	Temperate	Wild Harvested	(121)
37	*Astragalus membranaceus var. mongholicus*	Fabaceae			LC	Perennial	*Siberia to Russian Far East and W. & N. China*	Temperate	Wild Harvested	(122), (123)
38	Balanites aegyptiaca	Zygophyllaceae	*Balanites aegyptiaca* (L.) Delile		LC	Tree	Africa, Israel to Arabian Peninsula	desert or dry shrubland biome	Wild Harvested	(101)
39	*Barleria prionitis*	Acanthaceae	*Barleria prionitis* L.		LC	Shrub or Subshrub	NE. Tropical Africa, Madagascar, Tropical & Subtropical Asia	Wet tropical	Wild Harvested	(44)
40	Bauhinia cheilantha	Capparaceae	*Bauhinia cheilantha* (Bong.) Steud.		LC	Shrub or Tree	Bolivia to Brazil and Paraguay	Dry tropical	Wild Harvested	(112)
41	Bergenia ciliata	Saxifragaceae	*Bergenia ciliata* (Haw.) Sternb		LC	Perennial	W. Himalaya to SW. Nepal	Temperate	Wild Harvested	(52), (105)
42	Bergenia purpurascens	Saxifragaceae	*Bergenia purpurascens* (Hook.f. & Thomson) Engl.		Not Listed	Perennial	Central Himalaya to China (SW. Sichuan, N. Yunnan) and N. Myanmar	Subalpine or Subarctic	Wild Harvested	(52)
43	Bergenia stracheyi	Saxifragaceae	*Bergenia stracheyi* (Hook.f. & Thomson) Engl.		Not Listed	Perennial	E. Afghanistan to SW. Tibet and Nepal	Temperate	Wild Harvested	(52)
44	Blumea balsamifera	Asteraceae	*Blumea balsamifera* (L.) DC.		LC	Shrub	Tropical & Subtropical Asia	Temperate	Wild Harvested	(124), (90)

45	Boscia angustifolia	Capparaceae	*Boscia angustifolia* A.Rich.	Boscia integrifolia J.St.-Hil.	LC	Shrub or tree	S. Africa, Arabian Peninsula	desert or dry shrubland biome	Wild Harvested	(101)
46	*Boscia coriacea*	Capparaceae	*Boscia coriacea Graells*		Not Listed	Shrub or tree	NE. & E. Tropical Afric	Seasonally dry tropical biome	Wild Harvested	(101)
47	*Boswellia serrata*	Burseraceae	*Boswellia serrata* Roxb.		LC	Tree	Indian Subcontinent	Dry tropical	Wild Harvested	(49)
48	*Bowiea volubilis*	Asparagaceae	*Bowiea volubilis* Harv. ex T.Moore & Mast.		Not Listed	Bulbous	S. Africa	Dry tropical	Wild Harvested	(110)
49	*Bulbine frutescens*	Asphodelaceae	*Bulbine frutescens* (L.) Willd.		Not Listed	Bulbous Geophyte	S. Africa	Dry surbland	Wild Harvested	(71)
50	*Bulbophyllum odoratissimum*	Orchidaceae	*Bulbophyllum odoratissimum* (Sm.) Lindl. ex Wall.		Not Listed	Pseudobulbous lithophyte	*Nepal to S. China*	Wet tropical	Wild Harvested	(125)
51	*Caesalpinia bonduc*	Fabaceae	*Caesalpinia bonduc* (L.) Roxb		LC	Climbing shrub or Liana	*Tropics & Subtropics.*	Dry tropical	Wild Harvested	(126)
52	Caesalpinia sappan	Fabacea	*Caesalpinia sappan* L.		LC	Shrub or Tree	Indian Subcontinent to Indo-China.	Seasonally dry tropical biome	Wild Harvested	(90)
53	Calamintha nepeta subsp. Glandulosa	Lamiaceae	*Calamintha nepeta subsp. glandulosa* (Req.) P.W.Ball	*Clinopodium nepeta* subsp. *spruneri* (Boiss.) Bartolucci & F.Conti	Not Listed	Perennial	Great Britain, Medit. to Caucasus	Temperate	Wild Harvested	(51)
54	*Camptotheca acuminata*	Nyssaceae	*Camptotheca acuminata* Decne.		Not Listed	Shrub or Tree	*S. China to N. Vietnam*	*subtropical biome*	Cultivated and Wild Harvested	(98)
55	Capparis spinosa	Capparaceae	*Capparis spinosa* L.		LC	Shrub or Subshrub	Medit. to Mongolia and Indian Subcontinent, Philippines to W. & S. Pacific	subtropical biome	Wild Harvested	(127)

56	Carissa edulis	Apocynaceae	*Carissa edulis* (Forssk.) Vahl	Carissa spinarum L.	LC	Scrambling Shrub or Tree	Africa to Indo-China	Dry tropical	Wild Harvested	(101)
57	Castanopsis argentea	Fagaceae	*Castanopsis argentea* (Blume) A.DC.		EN	Tree	Arunachal Pradesh to W. Malesia	Wet tropical	Wild Harvested	(44)
58	*Cenostigma microphyllum*	Fabaceae	*Cenostigma microphyllum* (Mart. ex G.Don) Gagnon & G.P.Lewis		LC	Shrub or Tree	NE. Brazil, Paraguay	Dry tropical	Wild Harvested	(128)
59	*Cephalotaxus oliveri*	Cephalotaxaceae	*Cephalotaxus oliveri* Mast.		VU	Shrub	*S. China*	Temperate	Cultivated	(129)
60	Cereus Jamacaru	Cactaceae	*Cereus jamacaru* DC.		LC	Sucullent Tree	Central & E. Brazil	Dry tropical	Wild Harvested	(112)
61	*Chimonanthus grammatus*	Calycanthaceae	*Chimonanthus grammatus* M.C.Liu		Not Listed	Shrub or Tree	*China (Jiangxi: Anyuan)*	Temperate	Cultivated and Wild Harvested	(130)
62	*Chimonanthus nitens*	Calycanthaceae	*Chimonanthus nitens* Oliv.		LC	Shrub	*Central & S. China*	Temperate	Wild Harvested	(130)
63	*Chimonanthus praecox*	Calycanthaceae	*Chimonanthus praecox* (L.) Link		LC	Shrub	*Central & S. China*	Temperate	Wild Harvested	(130)
64	*Chimonanthus salicifolius*	Calycanthaceae	*Chimonanthus salicifolius* S.Y.Hu		Not Listed	Shrub	*China (Jiangxi, Anhui, Zhejiang)*	Temperate	Wild Harvested	(130)
65	*Chimonanthus zhejiangensis*	Calycanthaceae	*Chimonanthus zhejiangensis* M.C.Liu		Not Listed	Shrub	China (Zhejiang)	Temperate	Wild Harvested	(130)
66	*Cinnamomum camphora*	Lauraceae	*Cinnamomum camphora (L.)* Presl		LC	Tree	*Korea (Jeju-do), W. Central & S. Japan to E. & S. Taiwan*	*subtropical biome*	Cultivated	(130)
67	*Cinnamomum mairei*	Lauraceae	*Cinnamomum mairei* H. Lév		EN	Tree	*China (W. Sichuan, NE. Yunnan) to Vietnam*	*subtropical biome*	Wild Harvested	(131)
68	*Cirsium japonicum*	Asteraceae	*Cirsium japonicum* DC.		Not Listed	Perennial	*China to Vietnam, Temp. E. Asia*	Temperate	Cultivated and Wild Harvested	(132)
69	*Cistanche deserticola*	Orobanchaceae	*Cistanche deserticola* Ma		Not Listed	Holoparasitic Perennial	Mongolia to N. China	Temperate	Cultivated and Wild Harvested	(133)

70	*Citrus medica L. var. sarcodactylis*	Rutaceae	*Citrus medica* L. *var. sarcodactylis* Swingle		LC	Tree	china	subtropical biome	Cultivated	(134)
71	*Cleidion javanicum*	Euphorbiaceae	*Biancaea sappan* (L.) Tod.		LC	Tree	Asia to Vanuatu	Wet tropical	Wild Harvested	(47)
72	*Clerodendrum infortunatum*	Lamiaceae	*Clerodendrum infortunatum* L.		LC	Shrub	ndian Subcontinent to China (Yunnan) and Indo-China, Philippines	Wet tropical	Wild Harvested	(135)
73	*Clerodendrum serratum*	Lamiaceae	*Clerodendrum serratum* (L.) Moon	Rotheca serrata (L.) Steane & Mabb.	Not Listed	Shrub	Indian Subcontinent to S. China and W. Malesia	Wet tropical	Wild Harvested	(90)
74	*Clinopodium polycephalum*	Lamiaceae	*Clinopodium polycephalum* (Vaniot) C.Y.Wu & S.J.Hsuan		Not Listed	Perennial	Central & S. China.	Temperate	Wild Harvested	(136)
75	*Clivia miniata*	Amaryllidaceae	*Clivia miniata* (Lindl.) Verschaff.		Not Listed	Epiphyte	S. Africa	Subtropical	Wild Harvested	(22)
76	*Cnidium officinale*	Apiaceae	*Cnidium officinale* Makino		Not Listed	Tree	Japan	Temperate	Wild Harvested	(137)
77	*Comiphora africana*	Burseraceae	*Commiphora africana* (A.Rich.) Engl		Not Listed		Tropical & S. Africa	desert or dry shrubland biome	Wild Harvested	(101)
78	*Coptis chinensis*	Ranunculaceae	*Coptis chinensis* Franch.		Not Listed	Perennial Rhimatous	*Central & E. Central China*	Temperate	Cultivated	(138)
79	*Coptis deltoidea*	Ranunculaceae	*Coptis deltoidea* C.Y.Cheng & P.K.Hsiao		Not Listed	Perennial Rhimatous	*China (W. Sichuan*	Temperate	Wild Harvested	(138)
80	*Coptis teeta*	Ranunculaceae	*Coptis teeta* Wall.		EN	Perennial Rhimatous	*Arunachal Pradesh to China (NW. Yunnan) and N. Indo-China*	Temperate	Wild Harvested	(138)
81	*Cordia monoica*	Boraginaceae	*Cordia monoica* Roxb		LC	Shrub or Tree	Tropical & S. Africa, SW. Madagascar, SW. Arabian Peninsula, India	Seasonally dry tropical biome	Wild Harvested	(101)

							to Myanmar, S. Malesia			
82	*Crataegus azarolus*	Rosaceae	*Crataegus azarolus* L		LC	Shrub or Tree	N. Africa, E. Medit. to Iraq	Subtropical	Cultivated and Wild Harvested	(139)
83	Crataegus monogyna	Rosaceae	*Crataegus monogyna* Jacq		Not Listed	Tree	Europe to Caucasus, N. Africa to Iraq	Temperate	Wild Harvested	(139)
84	*Cremastra appendiculata*	Orchidaceae	*Cremastra appendiculata* (D.Don) Makino		Not Listed	Pseudobulbous lithophyte	*Central Himalaya to S. Sakhalin and Temp. E. Asia.*	*subtropical biome*	Wild Harvested	(140)
85	*Crepidium acuminatum*	Orchidaceae	*Crepidium acuminatum* (D.Don) Szlach.		Not Listed	Pseudobulbous geophyte or epiphyte	Indian Subcontinent to S. China and Philippines	Wet tropical	Wild Harvested	(141)
86	*Crocus sativus*	Iridaceae	*Crocus sativus subsp. cartwrightianus* (Herb.) K.Richt.		Not Listed	Tuberous Geophyte	Greece	Sub tropical	Cultivated	(142)
87	*Croton dichogamus*	Euphorbiaceae	*Croton dichogamus* Pax		LC	Shrub or Tree	Ethiopia to Mozambique	Seasonally dry tropical biome	Wild Harvested	(101)
88	*Croton megalocarpus*	Euphorbiaceae	*Croton megalocarpus* Hutch		LC	Tree	S. Somalia to S. Tropical Africa	Seasonally dry tropical biome	Wild Harvested	(101)
89	*Croton roxburghii*	Euphorbiaceae	*Croton roxburghii* N.P.Balakr.	Croton persimilis Müll.Arg.	LC	Shrub or tree	Indian Subcontinent to China (S. Yunnan) and Indo-China	Wet tropical	Wild Harvested	(47)
90	*Curcuma* longa	Zingiberaceae	*Curcuma longa* L.		Not Listed	Rhizomatous Geophyte	*cultigen from SW. India*	Dry tropical	Cultivated	(143)
91	*Cynara cardunculus*	Asteraceae	*Cynara cardunculus* L.		LC	Perennial	Macaronesia, Medit.	Temperate	Cultivated Wild Harvested	(74)
92	*Cynara tournefortii*	Asteraceae	*Cynara tournefortii* Boiss. & Reut.		Not Listed	Perennial	S. Portugal to Central & S. Spain, Morocco	subtropical biome	Wild Harvested	(45)
93	*Cypripedium japonicum*	Orchidaceae	*Cypripedium japonicum* Thunb.		EN	Rhizomatous Geophyte	Central & E. Central China, Central & S. Korea, Japan	Temperate	Cultivated and Wild Harvested	(144)

94	*Dactylorhiza hatagirea*	Orchidaceae	*Dactylorhiza hatagirea* (D.Don) Soó		EN	Tuberous Geophyte	Mongolia to Himalaya	Temperate	Wild Harvested	(106), (145)
95	*Daphne mucronata*	Thymelaeaceae	*Daphne mucronata* Royle		Not Listed	Shrub	SE. Türkiye to W. Himalaya and Arabian Peninsula	Temperate	Wild Harvested	(146), (147)
96	*Dendrobium acinaciforme*	Orchidaceae	*Dendrobium acinaciforme* Roxb.		Not Listed	Pseudobulbous epiphyte	E. Sulawesi (Kep. Banggai) to New Guinea (Kep. Aru).	Wet tropical	Cultivated	(54)
97	*Dendrobium aduncum*	Orchidaceae	*Dendrobium aduncum* Lindl.		Not Listed	Pseudobulbous epiphyte	*E. Himalaya to S. China*	Subtropical	Cultivated	(54)
98	*Dendrobium aphyllum*	Orchidaceae	*Dendrobium aphyllum* (Roxb.) C.E.C.Fisch.		LC	Pseudobulbous epiphyte	*Nepal to S. China and Peninsula Malaysia*	Wet tropical	Cultivated	(54)
99	*Dendrobium bellatulum*	Orchidaceae	*Dendrobium bellatulum* Rolfe		Not Listed	Pseudobulbous epiphyte	E. Himalaya to China (S. Yunnan) and Indo-China	Wet tropical	Cultivated	(54)
100	*Dendrobium cariniferum*	Orchidaceae	*Dendrobium cariniferum* Rchb.f.		Not Listed	Pseudobulbous Epiphyte	Assam to China (S. Yunnan) and Indo-China	Subtropical	Cultivated	(54)
101	*Dendrobium chrysanthum*	Orchidaceae	*Dendrobium chrysanthum* Wall. ex Lindl.		Not Listed	Pseudobulbous Epiphyte	*Himalaya to S. China and Indo-China*	Wet tropical	Cultivated	(54)
102	*Dendrobium chrysotoxum*	Orchidaceae	*Dendrobium chrysotoxum* Lindl.		Not Listed	Pseudobulbous Epiphyte	Arunachal Pradesh to China (Yunnan) and Indo-China	Wet tropical	Cultivated	(54)
103	*Dendrobium crepidatum*	Orchidaceae	*Dendrobium crepidatum* Lindl. & Paxton		Not Listed	Pseudobulbous Epiphyte	Himalaya to China (S. Yunnan, SW. Guizhou) and Indo-China	Subtropical	Cultivated	(54)
104	*Dendrobium devonianum*	Orchidaceae	*Dendrobium devonianum* Paxton		Not Listed	Pseudobulbous Epiphyte	Bhutan to S. Chin	Wet tropical	Cultivated	(54)
105	*Dendrobium falconeri*	Orchidaceae	*Dendrobium falconeri* Hook.		Not Listed	Pseudobulbous Epiphyte	*E. Sikkim to S. China and Indo-*	Wet tropical	Cultivated	(54)

							China, W. Taiwan			
106	*Dendrobium fimbriatum*	Orchidaceae	*Dendrobium fimbriatum* Dalzell		Not Listed	Pseudobulbous Epiphyte	*Himalaya to S. China and Indo-China*	Subtropical	Cultivated	(54)
107	*Dendrobium gibsonii*	Orchidaceae	*Dendrobium gibsonii* Paxton		Not Listed	Pseudobulbous Epiphyte	Central Himalaya to China (S. Yunnan, Guangxi) and Indo-China	Wet tropical	Cultivated	(54)
108	*Dendrobium hancockii*	Orchidaceae	*Dendrobium hancockii* Rolfe		Not Listed	Pseudobulbous Epiphyte	Central & S. China to N. Vietnam	Temperate	Cultivated	(54)
109	*Dendrobium hercoglossum*	Orchidaceae	*Dendrobium hercoglossum* Rchb.f.		Not Listed	Pseudobulbous Epiphyte	S. China to Peninsula Malaysia, Philippines (Mindanao	Wet tropical	Cultivated	(54)
110	*Dendrobium hookerianum*	Orchidaceae	*Dendrobium hookerianum* Lindl.		Not Listed	Pseudobulbous Epiphyte	Central Himalaya to China (W. Yunnan)	Subtropical	Cultivated	(54)
111	*Dendrobium huoshanense*	Orchidaceae	*Dendrobium huoshanense* Z.Z.Tang & S.J.Cheng		CR	Pseudobulbous Epiphyte	China (Henan, Anhui	Subtropical	Cultivated	(54)
112	*Dendrobium jenkinsii*	Orchidaceae	*Dendrobium jenkinsii* Wall. ex Lindl.		Not Listed	Pseudobulbous Epiphyte	E. Himalaya to China (S. Yunnan) and N. Indo-China	Subtropical	Cultivated and Wild Harvested	(54)
113	*Dendrobium leptocladum*	Orchidaceae	*Dendrobium leptocladum* Hayata		EN	Pseudobulbous Epiphyte	Central & S. Taiwan	Wet tropical	Cultivated and Wild Harvested	(54)
114	*Dendrobium linawianum*	Orchidaceae	*Dendrobium linawianum* Rchb.f.		Not Listed	Pseudobulbous Epiphyte	China (E. Guangxi), N. Taiwa	Subtropical	Cultivated	(54)
115	*Dendrobium lindleyi*	Orchidaceae	*Dendrobium lindleyi* Steud.		Not Listed	Pseudobulbous Epiphyte	*E. Himalaya to S. China and Indo-China*	Wet tropical	Cultivated	(54)

116	*Dendrobium loddigesii*	Orchidaceae	*Dendrobium loddigesii* Rolfe		Not Listed	Pseudobulbous Epiphyte	S. China to Indo-China	Wet tropical	Cultivated	(54)
117	*Dendrobium moniliforme*	Orchidaceae	*Dendrobium moniliforme (L.) Sw.*		Not Listed	Pseudobulbous Epiphyte	Himalaya to Temp. E. Asia.	Temperate	Cultivated	(54, 148), (149)
118	*Dendrobium nobile*	Orchidaceae	*Dendrobium nobile* Lindl.		Not Listed	Pseudobulbous Epiphyte	*Nepal to S. China and Indo-China*	*subtropical biome*	Cultivated	(54, 148), (149)
119	*Dendrobium officinale*	Orchidaceae	*Dendrobium officinale* Kimura & Migo		CR	Pseudobulbous Epiphyte	S. China to N. Vietnam, S. Japan to E. Central Taiwan	Subtropical	Cultivated	(148), (54)
120	*Dendrobium parishii*	Orchidaceae	*Dendrobium parishii* H.Low		Not Listed	Pseudobulbous Epiphyte	Arunachal Pradesh to China (SE. Yunnan, Guizhou) and Indo-China	Wet tropical	Cultivated	(54)
121	*Dendrobium pendulum*	Orchidaceae	*Dendrobium pendulum* Roxb.		Not Listed	Pseudobulbous Epiphyte	Sikkim to China (S. Yunnan) and Indo-China	Wet tropical	Cultivated	(54)
122	*Dendrobium primulinum*	Orchidaceae	*Dendrobium primulinum* Lindl.		Not Listed	Pseudobulbous Epiphyte	Himalaya to China (S. Yunnan) and Indo-China	Subtropical	Cultivated	(54)
123	*Dendrobium strongylanthum*	Orchidaceae	*Dendrobium strongylanthum* Rchb.f.		Not Listed	Pseudobulbous Epiphyte	China (Yunnan) to N. Indo-China, Hainan	Subtropical	Wild Harvested	(54)
124	*Dicksonia blumei*	Dicksoniaceae	*Dicksonia blumei* (Kunze) T.Moore		Not Listed	Tree	Sumatera to Lesser Sunda Islands (Bali)	Wet tropical	Wild Harvested	(44)
125	*Dioscorea elephantipes*	Dioscoreaceae	*Dioscorea elephantipes* (L'Hér.) Engl.		LC	Climbing Caudex	Cape Prov.	subtropical biome	Wild Harvested	(110)
126	*Dipterocarpus baudii*	Dipterocarpaceae	*Dipterocarpus baudii* Korth.		VU	Tree	Bangladesh to Sumatera	Wet tropical	Wild Harvested	(44)
127	*Echinacea purpurea*	Asteraceae	*Echinacea purpurea* (L.) Moench		LC	Perennial	Central & E. U.S.A.	Temperate	Cultivated and Wild Harvested	(74)

128	*Echinops kebericho* Mesfin	Asteraceae	*Echinops kebericho* Mesfin		NT	Shrub	*ethiopia*	Dry tropical	Wild Harvested	(150)
129	*Ephedra equisetina*	Ephedraceae	*Ephedra equisetina* Bunge		Not Listed	Shrub	Caucasus to S. Russian Far East	Temperate	Wild Harvested	(151)
130	*Ephedra gerardiana*	Ephedraceae	*Ephedra gerardiana* Wall. ex Klotzsch & Garcke		VU	Subshrub	Afghanistan to SW. Siberia and Himalaya	Subalpine or Subarctic	Wild Harvested	(152)
131	*Ephedra intermedia*	Ephedraceae		Zanthoxylum tingoassuiba A.St.-Hil.	LC	Subshrub	*Iran to Mongolia and Himalaya*	Temperate	Cultivated and Wild Harvested	(153), (151)
132	*Ephedra sinica*	Ephedraceae	*Ephedra sinica* Stapf		LC	Subshrub	S. Siberia to N. & NE. China	Temperate	Cultivated and Wild Harvested	(153), (151), (154), (50)
133	*Eriocaulon buergerianum* Körnicke	Eriocaulaceae	*Eriocaulon buergerianum* Körnicke		*Not Listed*	Perennial	*E. Asia to Vietnam.*	*subtropical biome*	Wild Harvested	(155)
134	*Erythrina Velutina*	Fabaceae	*Erythrina velutina* Willd.		LC	Tree	Caribbean to S. Tropical America	Dry tropical	Wild Harvested	(112)
135	*Euchresta horsfieldii*	Fabaceae	*Euchresta horsfieldii* (Lesch.) Benn.		Not Listed	Shrub	Assam to China (SE. Yunnan) and W. & Central Malesia	Wet tropical	Wild Harvested	(44)
136	*Euclea divinorum*	Ebenaceae	*Euclea divinorum* Hiern		LC	Shrub or Tree	Ethiopia to S. Africa	Dry tropical	Wild Harvested	(101)
137	*Eucommia ulmoides*	Eucommiaceae	*Eucommia ulmoides* Oliv.		VU	Tree	*Central & S. China.*	Temperate	Cultivated	(156)
138	*Eurycoma longifolia*	Simaroubaceae	*Eurycoma longifolia* Jack		LC	Tree	Indo-China to W. Malesia	Wet tropical	Wild Harvested	(44)
139	*Euscaphis japonica*	Staphyleaceae	*Euscaphis japonica* (Thunb.) Kanitz		LC	Tree	Central & S. China to N. Vietnam and Temp. E. Asia	subtropical biome	Wild Harvested	(157)
140	*Eusideroxylon zwageri*	Lauraceae	*Eusideroxylon zwageri* Teijsm. & Binn.		VU	Tree	Sumatera, Borneo	Wet tropical	Wild Harvested	(44)

141	*Forsythia suspensa* (Thunb.) Vahl	Oleaceae	*Forsythia suspensa* (Thunb.) Vahl		Not Listed	Shrub	*China*	Temperate	Wild Harvested	(158)
142	*Fritillaria cirrhosa*	Liliaceae	*Fritillaria cirrhosa* D.Don		VU	Bulbous Geophyte	*Pakistan to Central China*	Temperate	Wild Harvested	(104) (159) (29) (160)
143	*Fritillaria delavayi*	Liliaceae	*Fritillaria delavayi* Franch.		Not Listed	Bulbous Geophyte	Sikkim to S. Central China	Temperate	Wild Harvested	(103)
144	*Fritillaria imperialis*	Liliaceae	*Fritillaria imperialis* L.		Not Listed	Bulbous Geophyte	E. Central & SE. Türkiye to W. Himalaya	Temperate	Wild Harvested	(161)
145	*Fritillaria przewalskii*	Liliaceae	*Fritillaria przewalskii* Maxim. ex Batalin		Not Listed	Bulbous Geophyte	E. Qinghai to China (Sichuan, S. Gansu)	Temperate	Wild Harvested	(103)
146	*Fritillaria unibracteata*	Liliaceae	*Fritillaria unibracteata* P.K.Hsiao & K.C.Hsia	Not Listed	Not Listed	Bulbous Geophyte	SE. Qinghai to China (Sichuan, S. Gansu	Temperate	Wild Harvested	(103)
147	*Garcinia indica*	Clusiaceae	*Garcinia indica* (Thouars) Choisy		VU	Tree	W. & SW. India	Dry tropical	Cultivated	(162)
148	*Gastrodia elata*	Orchidaceae	*Gastrodia elata* Blume		VU	Holomycotrop hic rhizomatous geophyte	Himalaya to Russian Far East and Temp. E. Asia.	Temperate	Cultivated Wild Harvested	(163)
149	*Gentiana boryi*	Gentianaceae	*Gentiana boryi* Boiss.		Not Listed	Perennial	Spain (Cord. Cantábrica, Sierra de Gredos, Sierra Nevada)	Subalpine or Subarctic	Wild Harvested	(45)
150	*Gentiana clusii*	Gentianaceae	*Gentiana clusii* Perr. & Songeon		Not Listed	Perennial	Spain (Cord. Cantábrica, Sierra de Gredos, Sierra Nevada)	Subalpine or Subarctic	Wild Harvested	(45)
151	*Gentiana lhassica*	Gentianaceae	*Gentiana lhassica* Burkill		Not Listed	perennial	SW. Qinghai, E. Tibet	Subalpine or Subarctic	Wild Harvested	(103)
152	*Gentiana macrophylla*	Gentianaceae	*Gentiana macrophylla* Bertol.		Not Listed	Perennial	Siberia to Central China	Temperate	Wild Harvested	(164)

153	*Gentiana manshurica*	Gentianaceae	*Gentiana manshurica Kitag.*		Not Listed	Perennial	*S. Russian Far East to China, Taiwan*	Temperate	Wild Harvested	(165)
154	Gentiana quadrifaria	Gentianaceae	*Gentiana quadrifaria* Blume		Not Listed	Perennial	Jawa (Mountains)	Temperate	Wild Harvested	(44)
155	Gentiana rhodantha	Gentianaceae	*Gentiana rhodantha* Franch.		Not Listed	Shrub	Central & E. Central China	Temperate	Wild Harvested	(166)
156	*Gentiana rigescens*	Gentianaceae	*Gentiana rigescens* Franch.		Not Listed	Subshrub	*Myanmar to S. Central China*	Temperate	Cultivated and Wild Harvested	(75) (167)
157	*Gentiana scabra*	Gentianaceae	*Gentiana scabra* Bunge		EN	Perennial	SE. Siberia to Japan and N. & E. China	Temperate	Wild Harvested	(100)
158	*Gentiana sierrae*	Gentianaceae	Gentiana sierrae Briq.		EN	Perennial	Spain (Sierra Nevada), Morocco (Djebel Toubkal)	Subalpine or Subarctic	Wild Harvested	(45)
159	*Ginkgo biloba*	Ginkgoaceae	*Ginkgo biloba* L.		EN	tree	China (Zhejiang)	Temperate	Cultivated and Wild Harvested	(168) (169)
160	*Glycyrrhiza aspera*	Fabaceae	*Glycyrrhiza aspera* Pall.		Not Listed	Perennial	S. European Russia to Xinjiang	Temperate	Cultivated and Wild Harvested	(170)
161	Glycyrrhiza glabra L.	Fabaceae	*Glycyrrhiza glabra* L.		LC	Perennial	Central Medit. to Mongolia and Pakistan	Temperate	Cultivated and Wild Harvested	(170)
162	*Glycyrrhiza inflata*	Fabaceae	*Glycyrrhiza inflata* Batalin.		Not Listed	Perennial	China to Mongolia	Temperate	Cultivated and Wild Harvested	(170)
163	Glycyrrhiza pallidiflora	Fabaceae	*Glycyrrhiza pallidiflora* Maxim		Not Listed	Perennial	Russian Far East to China	Temperate	Cultivated and Wild Harvested	(170)
164	*Glycyrrhiza squamulosa*	Fabaceae	*Glycyrrhiza squamulosa* Franch		Not listed	Perennial	Mongolia to N. & E. China	Temperate	Wild Harvested	(170)
165	*Glycyrrhiza yunnanensis*	Fabaceae	*Glycyrrhiza yunnanensis* S.H.Cheng & L.K.Tai ex P.C.Li		Not Listed	Perennial	China (Yunnan)	Temperate	Wild Harvested	(170)

166	*Gymnadenia orchidis*	Orchidaceae	*Gymnadenia orchidis* Lindl.		Not Listed	Tuberous geophyte	*N. Pakistan to Central China and Myanmar, Central India (Satpura Range)*	Temperate	Wild Harvested	(171)
167	*Gynostemma pentaphyllum*	Cucurbitaceae	*Gynostemma pentaphyllum* (Thunb.) Makino		Not Listed	Climber	Kuril Islands to Tropical & Subtropical Asia	subtropical biome	Wild Harvested	(70)
168	*Harrisonia abyssinica*	Rutaceae	*Harrisonia abyssinica* Oliv.		LC	Shrub or Tree	Tropical Africa	Seasonally dry tropical biome	Wild Harvested	(101)
169	*Hedeoma multiflora*	Lamiaceae	*Hedeoma multiflora* Benth.		Not Listed	Subshrub	SE. & S. Brazil to Central Argentina	subtropical biome	Wild Harvested	(99)
170	*Helleborus odorus subsp. cyclophyllus*	Ranunculaceae	*Helleborus cyclophyllus* (A.Braun) Boiss.		Not Listed	Perennial	Balkan Peninsula	Temperate	Wild Harvested	(172)
171	*Homonoia riparia*	Euphorbiaceae	*Homonoia riparia* Lour.		LC	Shrub	Tropical & Subtropical Asia	Wet tropical	Wild Harvested	(173) (174)
172	*Houttuynia cordata*	Saururaceae	*Houttuynia cordata* Thunb		Not Listed	Rhizomatous geophyte	Himalaya to Temp. E. Asia and Indo-China	Temperate	Cultivated and wild harvested	(175)
173	*Hylomecon japonica*	Papaveraceae	*Hylomecon japonica (Thunb.)* Prantl		Not Listed	Perennial	*Central & E. Central China, Japan (Honshu)*	Temperate	Wild Harvested	(176)
174	Hypericum coadunatum	Hypericaceae	*Hypericum coadunatum* C.Sm. ex Link		Not Listed	Subshrub	Canary Islands (Gran Canaria)	subtropical biome	Wild Harvested	(45)
175	*Hypericum perforatum*	Hypericaceae	*Hypericum perforatum* L.		LC	Perennial	*Europe to China, NW. Africa*	Temperate	Cultivated and Wild Harvested	(74), (47)
176	*Hypericum roberti*	Hypericaceae	*Hypericum roberti* Coss. ex Batt.		Not Listed	Subshrub	NE. Algeria to NW. Tunisia	subtropical biome	Wild Harvested	(45)
177	*Inula cappa*	Asteraceae	*Inula cappa* (Buch.-Ham. ex D.Don) DC.	Duhaldea cappa (Buch.-Ham. ex	Not Listed	Shrub or tree	Pakistan to S. China and Jawa.	Dry tropical	Wild Harvested	(47)

				D.Don) Pruski & Anderb.						
178	*Juniperus procera*	Cupressaceae	*Juniperus procera* Hochst. ex Endl.		LC	Tree	Eritrea to Zimbabwe, SW. Arabian Peninsula	Seasonally dry tropical biome	Wild Harvested	(177)
179	*Kigelia africana*	Bignoniaceae	*Kigelia africana* (Lam.) Benth.		LC	Tree	Tropical & S. Africa	Dry tropical	Wild Harvested	(24)
180	*Lallemantia iberica*	Lamiaceae	*Dracocephalum royleanum* Benth.		Not Listed		Caucasus to W. & Central Asia.	Temperate	Cultivated and Wild Harvested	(24)
181	*Lallemantia royleana*	Lamiaceae	*Lallemantia royleana (Benth.) Benth.*		Not Listed		Caucasus to W. Himalaya and Arabian Peninsula.	Temperate	Wild Harvested	(118)
182	Lamium album	Lamiaceae	*Lamium album* L.		Not Listed	Perennial	Temp. Eurasia	Temperate	Wild Harvested	(178)
183	*Laurus nobilis*	Lauraceae	*Laurus nobilis* L.		LC	*tree*	*Laurus nobilis*	*subtropical biome*	Cultivated and Wild Harvested	(45)
184	*Lavandula buchii*	Lamiaceae	*Lavandula buchii* Webb & Berthel.		Not Listed	Subshrub	Canary Islands (Tenerife	subtropical biome	Wild Harvested	(68)
185	*Lavandula latifolia.*	Lamiaceae	*Lavandula latifolia* Medik.		LC	subshrub	Spain to N. Central Italy	Temperate	Wild Harvested	(179)
186	*Lebrunia bushaie*	Clusiaceae	*Lebrunia bushaie* Staner		VU	tree	Central DR Congo	Wet tropical	Wild Harvested	(71)
187	*Leonurus japonicus*	Lamiaceae	*Leonurus macranthus* Maxim.		Not Listed	*Subshrub*	*China to Russian Far East and N. Australia*	*subtropical biome*	Wild Harvested	(180) (181)
188	*Ligusticum chuanxiong*	Apiaceae	*Ligusticum chuanxiong* S.H.Qiu, Y.Q.Zeng, K.Y.Pan, Y.C.Tang & J.M.Xu		Not Listed	*China South-Central*	Temperate	*Ligusticum chuanxiong*	Cultivated	(182)
189	*Lilium polyphyllum*	Liliaceae	*Lilium polyphyllum* D.Don		Not Listed	*bulbous geophyte*	*Afghanistan to N. India*	Temperate	Wild Harvested	(183)
190	*Lippia javanica*	Verbenaceae	*Lippia javanica* (Burm.f.) Spreng.		Not Listed		Ethiopia to S. Africa.	Dry tropical	Wild Harvested	(62)

191	*Liriodendron chinense*	Magnoliaceae	*Liriodendron chinense* (Hemsl.) Sarg.		NT	*tree*	*Central & S. China to N. Vietnam*	Temperate	Wild Harvested	(184)
192	*Litsea glutinosa*	Lauraceae	*Litsea glutinosa* (Lour.) C.B.Rob.		Not Listed		*Tropical & Subtropical Asia*	Dry tropical	Wild Harvested	(185)
193	Lomatogoniopsis alpina	Gentianaceae	*Lomatogoniopsis alpina* T.N.Ho & S.W.Liu		Not Listed		NE. Tibet to China (NW. Sichuan	Subalpine or Subarctic	Wild Harvested	(103)
194	Lycium barbarum	Solanaceae	*Lycium barbarum* L		Not Listed	Shrub	N. & Central China	Temperate	Cultivated	(31)
195	Macaranga griffithiana	Euphorbiaceae	*Macaranga griffithiana* Müll.Arg.		LC	Tree	Indo-China to Sumatera	Wet tropical	Wild Harvested	(44)
196	*Magnolia biondii*	Magnoliaceae	*Magnolia biondii* Pamp.		LC	*tree*	*Central China.*	Temperate	Cultivated and wild Harvested	(186)
197	*Magnolia officinalis* subsp. *officinalis*	Magnoliaceae	*Magnolia officinalis* subsp. officinalis		Not Listed	*tree*	*China*	Temperate	Cultivated	(187)
198	*Matricaria chamomilla*	Asteraceae	*Matricaria chamomilla L.*		Not Listed	Perennial	Macaronesia, N. Africa, Temp. Eurasia to Indo-China	Temperate	Wild Harvested	(74) (187)
199	Maytenus Rigida	Celastraceae	*Maytenus rigida* Mart.		LC	Tree	Brazil	Dry tropical	Wild Harvested	(112)
200	Meconopsis aculeata	Papaveraceae	*Meconopsis aculeata* Royle			Monocarpic perennial	W. Himalaya to SW. Tibet	Subalpine or Subarctic	Wild Harvested	(104) (188)
201	*Meconopsis punicea*	Papaveraceae	*Meconopsis punicea* Maxim.		LC	Perennial	*S. & SE. Qinghai to China (SW. Gansu, NW. & W. Sichuan)*	Subalpine or Subarctic	Cultivated and Wild Harvested	(189)
202	*Melia azedarach*	Meliaceae	*Melia azedarach* L.		LC	Tree	Tropical & Subtropical Asia to N. & E. Australia	Dry tropical	Cultivated and Wild Harvested	(67)
203	*Melissa officinalis*	Lamiaceae	*Clinopodium nepeta subsp. spruneri* (Boiss.) Bartolucci & F.Conti		LC		Medit. to Central Asia	Temperate	Cultivated and Wild Harvested	(51)
204	*Mentha pulegium*	Lamiaceae	*Mentha pulegium* L.		LC	Perennial	*Macaronesia, Europe, Medit.*	Temperate	Cultivated	(21, 190)

							to N. Iran and N. Ethiopia			
205	*Mentha spicata*	Lamiaceae	*Mentha spicata* L.		LC	Perennial	Europe to China.	Temperate	Wild Harvested	(142)
206	*Micromeria juliana*	Lamiaceae	*Micromeria juliana* (L.) Benth. ex Rchb.		Not Listed	Subshrub	Mediterania	Subtropical	Wild Harvested	(51)
207	*Mondia whitei*	Apocynaceae	*Mondia whitei* (Hook.f.) Skeels		Not Listed	Tuberous	S. Africa.	Dry tropical	Wild Harvested	(57)
208	*Monotheca buxifolia*	Sapotaceae	*Monotheca buxifolia* (Falc.) A.DC.	Sideroxylon mascatense (A.DC.) T.D.Penn.	Not Listed	Shrub	S. Ethiopia to NW. & N. Pakistan	Subtropical	Wild Harvested	(191)
209	*Morinda officinalis*	Rubiaceae	*Morinda officinalis* F.C.How	*Gynochthodes officinalis* (F.C.How) Razafim. & B.Bremer	Not Listed	Climber	SE. China to Hainan	Wet tropical	Cultivated and Wild Harvested	(192)
210	*Moringa oleifera*	Moringaceae	*Moringa oleifera* Lam.		LC	Tree	NE. Pakistan to NW. India	Dry tropical	Cultivated	(193)
211	*Moringa stenopetala*	Moringaceae	*Moringa stenopetala* (Baker f.) Cufod.		DD	Tree	SW. & S. Ethiopia to Kenya	seasonally dry tropical biome.	Cultivated	(193)
212	*Mussaenda sanderiana*	Rubiacea	*Mussaenda sanderiana* Ridl.		Not Listed	Shrub	China (S. Yunnan) to Indo-China	Wet tropical	Wild Harvested	(47)
213	*Myracrodruon urundeuva*	Convolvulaceae	*Myracrodruon urundeuva* Allemão		LC	Tree	Bolivia to Brazil and N. Argentina	Dry tropical	Wild Harvested	(112)
214	*Myrcia tomentosa*	Myrtaceae	*Myrcia tomentosa* (Aubl.) DC.		LC	Shrub or Tree	Panama to S. Tropical America, Trinidad	Dry tropical	Wild Harvested	(194)
215	*Myristica dactyloides*	Myristicaceae	*Myristica dactyloides* Gaertn		VU	Tree	*Sri Lanka*	Wet tropical	Wild Harvested	(195)

216	*Myrsine africana*	Primulaceae	*Myrsine africana* L.		Not Listed	Shrub or Tree	Azores, Eritrea to S. Africa, Arabian Peninsula to China, Taiwan.	Sub Tropical	Cultivated and Wild Harvested	(72)
217	*Myrtus communis*		*Myrtus communis* L.		LC		Macaronesia to Pakistan	Sub Tropical	Wild Harvested	(72)
218	*Nardostachys jatamansi*	Caprifoliaceae	*Nardostachys jatamansi* (D.Don) DC.		CR	Perennial	Himalaya to W. & Central China and N. Myanmar	Temperate	Wild Harvested	(105), (106), (196), (69, 197)
219	*Neocalyptrocalyx longifolium*	Convolvulaceae	*Neocalyptrocalyx longifolium* (Mart.) Cornejo & Iltis		Not Listed	Shrub	NE. Brazil	Wet tropical	Wild Harvested	(112)
220	*Neopicrorhiza scrophulariiflora*	Scrophulariaceae	*Morisonia longifolia* (Mart.) Christenh. & Byng		Not Listed	Perennial	Himalaya to China (W. Sichuan, NW. Yunnan)	Temperate	Wild Harvested	(105, 106)
221	*Nepenthes reinwardtiana*	Nepenthaceae	*Nepenthes reinwardtiana* Miq.		LC	Climbing shrub	W. Sumatera, Borneo	Wet tropical	Wild Harvested	(44)
222	*Nepeta crispa*	Lamiaceae	*Nepeta crispa* Willd.		Not Listed	Perennial	Iran	Temperate	Wild Harvested	(43)
223	*Nepeta glomerulosa*	Lamiaceae	*Nepeta glomerulosa* Boiss.		Not Listed	Perennial	Iran to SW. Afghanistan	Temperate	Wild Harvested	(198)
224	*Notopterygium incisum*	Apiaceae	*Notopterygium incisum* K.C.Ting ex H.T.Chang	*Hansenia weberbaueriana* (Fedde ex H.Wolff) Pimenov & Kljuykov	Not Listed	Annual	NE. Tibet to Central China	Temperate	Cultivated and Wild Harvested	(103), (199)
225	*Ocimum basilicum*	Lamiaceae	*Ocimum basilicum* L.		Not Listed	Shrub	Tropical & Subtropical Asia to N. Australia	Dry tropical	Cultivated	(76)

226	*Olea africana*	Oleaceae	*Olea europaea* subsp. *africana* (Mill.) P.S.Green		Not Listed	Shrub or tree	Eritrea to S. Africa	Dry tropical	Wild Harvested	(101)
227	*Olea europaea*	Oleaceae	*Olea europaea* L.		DD	Shrub or Tree	Africa, Medit. to S. Central China	Subtropical	Cultivated	(23)
228	*Operculina hamiltonii*	Fabaceae	*Operculina hamiltonii* (G.Don) D.F.Austin & Staples		Not Listed	Climbing herbaceous	SE. Mexico to Tropical America	Dry tropical	Wild Harvested	(112)
229	*Operculina macrocarpa*	Fabaceae	*Operculina hamiltonii* (G.Don) D.F.Austin & Staples		Not Listed	Perennial	Central & E. Central U.S.A. to Mexico (Coahuila)	Temperate	Wild Harvested	(112)
230	*Origanum dictamnus*	Lamiaceae	*Origanum dictamnus* L.		NT	Subshrub	Kriti.	Subtropical	Wild Harvested	(51)
231	*Origanum onites*	Lamiaceae	*Origanum onites* L.		LC	Subshrub	Sicilia to Türkiye	Subtropical	Cultivate d and Wild Harvested	(51)
232	*Origanum vulgare subsp. Hirtum*	Lamiaceae	*Origanum vulgare* subsp. *hirtum* (Link) A.Terracc.		Not Listed	Perennial	SE. Europe to Türkiye	Temperate	Cultivated and Wild Harvested	(51)
233	*Oroxylum indicum*	Bignoniaceae	*Oroxylum indicum* (L.) Kurz		LC	Tree	S. China to Tropical Asia	Wet tropical	Wild Harvested	(200)
234	*Osmanthus fragrans*	Oleaceae	*Osmanthus fragrans* Lour.		LC	Shrub or Tree	Himalaya to S. Japan	Subtropical	Cultivated and Wild Harvested	(201)
235	*Paeonia delavayi*	Paeoniaceae	*Paeonia delavayi* Franch.		Not Listed	Shrub	E. Tibet to China (W. Sichuan, Yunnan)	Temperate	Wild Harvested	(202)
236	*Paeonia ostii*	Paeoniaceae	*Paeonia ostii* T.Hong & J.X.Zhang		Not Listed	Shrub	China (W. Henan to Anhui)	Temperate	Cultivated and Wild Harvested	(203)

237	*Paeonia rockii*	Paeoniaceae	*Paeonia rockii* (S.G.Haw & Lauener) T.Hong & J.J.Li ex D.Y.Hong		Not Listed	Shrub	China	Temperate	Cultivated and Wild Harvested	(201)
238	*Paeonia veitchii*	Paeoniaceae	*Paeonia veitchii Lynch*		Not Listed	Perennial	E. Tibet to N. & Central China	Tempere	Wild Harvested	(204)
239	*Panax japonicus*	Araliaceae	*Panax japonicus* (T.Nees) C.A.Mey.		Not Listed	Perennial	Korea, Japan	Temperate	Cultivated and Wild Harvested	(205)
240	*Panax japonicus var. major*	Araliaceae	*Panax japonicus* var. *major* (Burkill) C.Y.Wu & Feng		Not Listed	Rhizomatous geophyte	Central & E. Himalaya to Central & E. Central China	Temperate	Cultivated and Wild Harvested	(205)
241	*Panax notoginseng*	Araliaceae	*Panax notoginseng* (Burkill) F.H.Chen		Not Listed	Perennial	*China (SE. Yunnan) to N. Vietnam*	Temperate	Cultivated and Wild Harvested	(205)
242	*Panax pseudoginseng*	Araliaceae	*Panax pseudoginseng* Wall.		Not Listed	Perennial or rhizomatous geophyte	S. Tibet to Central Nepal	Temperate	Cultivated and Wild Harvested	*(46)*
243	*Panax quinquefolius*	Araliaceae	*Panax quinquefolius* L.		Not Listed	Perennial	*SE. Canada to N. Central & E. U.S.A*	Temperate	Cultivated and Wild Harvested	*(48, 206)*
244	*Panax stipuleanatus*	Araliaceae	*Panax stipuleanatus* H.T.Tsai & K.M.Feng		Not Listed	Rhizomatous geophyte	China (SE. Yunnan) to N. Vietnam	Temperate	Wild Harvested	(205)
245	*Panax zingiberensis*	Araliaceae	*Panax zingiberensis* C.Y.Wu & Feng		EN	Rhizomatous geophyte	China (SE. Yunnan) to N. Vietnam	Temperate	Wild Harvested	(205)
246	*Paris polyphylla*	Melanthiaceae	*Paris polyphylla* Sm.		VU	Rhizomatous geophyte	Himalaya to Central China and N. Myanmar	Temperate	Wild Harvested	(105, 106, 145, 207)
247	*Pedicularis longiflora*	Orobanchaceae	*Pedicularis longiflora* Rudolph		Not Listed	Shrub	Central Asia to S. Siberia and W. & Central China	Subalpine or subarctic	Wild Harvested	(208)

248	*Pellionia scabra*	Urticaceae	*Pellionia scabra* Benth.	*Pellionia pellucida* (Raf.) Merr.	Not Listed	Perennial	S. China to N. Vietnam, S. Central & S. Japan to Taiwan, Korea (Cheju Do)	Subtropical	Wild Harvested	(209)
249	*Pentatropis spiralis*	Apocynaceae	*Pentatropis spiralis* (Forssk.) Decne.	*Vincetoxicum spirale* (Forssk.) D.Z.Li	Not Listed	Shrub	Eritrea, Yemen	Desert or dry shrubland	Wild Harvested	(210)
250	*Phellodendron amurense*	Rutaceae	*Phellodendron amurense* Rupr.		DD	Tree	*Russian Far East to N. & E. China, Temp. E. Asia*	Temperate	Wild Harvested	(211)
251	*Phlogacanthus curviflorus*	Acanthaceae	*Phlogacanthus curviflorus* (Nees) Nees		Not Listed	Shrub or tree	Indian Subcontinent to China (Yunnan) and Indo-China	Wet tropical	Wild Harvested	(47)
252	*Phytolacca acinosa*	Phytolaccaceae	*Phytolacca acinosa* Roxb.		Not Listed	Perennial	N. Pakistan to Temp. E. Asia	Temperate biome	Wild Harvested	(118)
253	*Picrorhiza kurroa*	Plantaginaceae	*Picrorhiza kurroa* Royle ex Benth.		EN	Perennial	NE. Pakistan to W. Himalaya	Subalpine or subarctic	Wild Harvested	(212, 213)
254	*Pilocarpus microphyllus*	Rutaceae	*Pilocarpus microphyllus* Stapf ex Wardlew.		VU	Tree	N. & NE. Brazil.	Wet tropical	Wild Harvested	(214)
255	*Pinellia ternata*	Araceae	*Pinellia ternata* (Thunb.) Makino		Not Listed	Tuberous geophyte	China to Temp. E. Asia	Temperate	Wild Harvested	(215)
256	*Pinus merkusii*	Pinaceae	*Pinus merkusii* Jungh. & de Vriese		VU	Tree	N. Sumatera, Philippines (Luzon, Mindoro)	Wet tropical	Wild Harvested	(44)
257	*Pistacia eurycarpa*	Anacardiaceae	*Pistacia eurycarpa Yalt*		LC	Shrub or Tree	E. Türkiye to Afghanistan	Subtropical	Wild Harvested	(216)
258	*Pistacia khinjuk*	Anacardiaceae	*Pistacia khinjuk* Stocks		LC	Shrub or Tree	E. Medit. to Central Himalaya and	Subtropical	Wild Harvested	(216)

							Arabian Peninsula			
259	*Podophyllum hexandrum*	Berberidaceae	*Podophyllum hexandrum* Royle		EN	Perennial or rhizomatous geophyte	NE. Afghanistan to Central China	Temperate	Wild Harvested	(118)
260	*Polygala tenuifolia*	Polygalaceae	*Polygala tenuifolia* Willd.		Not Listed	Perennial	*Siberia to China and Korea*	Temperate	Cultivated and Wild Harvested	(217)
261	*Polygonatum kingianum*	Asparagaceae	*Polygonatum kingianum* Collett & Hemsl.		Not Listed	Rhizomatous geophyte	China South-Central, China Southeast, Myanmar, Thailand, Vietnam	Temperate	Cultivated and Wild Harvested	(218)
262	*Pomatosace filicula*	Primulaceae	*Pomatosace filicula* Maxim.	*Androsace filicula* (Maxim.) Heng C.Wang & Jiao Sun	Not Listed	Annual or biennial	NE. Tibet to China (NW. Sichuan)	Subalpine or subarctic	Wild Harvested	(219)
263	*Populus tremuloides*	Salicaceae	*Populus tremuloides* Michx.		LC	Tree	America to Mexico.	Temperate	Wild Harvested	(220)
264	*Prunus africana*	Rosaceae	*Prunus africana* (Hook.f.) Kalkman		VU	Tree	Ghana to Ethiopia and S. Africa, Comoros, Madagascar	Tropical	Wild Harvested	(96, 221, 222)
265	*Pterocarpus marsupium*	Fabaceae	*Pterocarpus marsupium* Roxb.		NT	Tree	Indian Subcontinent	Temperate	Cultivated and Wild Harvested	(223)
267	*Rauvolfia serpentina*	Apocynaceae	*Rauvolfia serpentina* (L.) Benth. ex Kurz		LC	Shrub	Indian Subcontinent to S. Central China and W. Malesia	Wet tropical	Wild Harvested	(44)

268	*Rheum nanum*	Polygonaceae	*Rheum nanum* Lingelsh.	*Koenigia hookeri* (Meisn.) T.M.Schust. & Reveal	Not Listed	Perennial	Central Asia to Mongolia and N. China.	Temperate	Wild Harvested	(224)
269	*Rheum tanguticum*	Polygonaceae	*Rheum tanguticum* (Maxim. ex Regel) Balf.		Not Listed	Perennial	Tibet to N. Central China	Temperate	Wild Harvested	(103)
270	*Rheum webbianum*	Polygalaceae	*Rheum webbianum* Royle		Not Listed	Perennial	Pakistan to SW. Tibet and W. Nepal	Subalpine or subarctic	Wild Harvested	(118, 225)
271	*Rhodiola alterna*	Crassulaceae	*Rhodiola alterna* S.H.Fu	*Rhodiola chrysanthemifolia* subsp. *sacra* (Raym.-Hamet) H.Ohba	Not listed	Perennial	SE. Qinghai to E. & S. Tibet and Central & E. Himalaya	Subalpine or subarctic	Wild Harvested	(103)
272	*Rhodiola chrysanthemifolia*	Crassulaceae	*Rhodiola chrysanthemifolia* (H.Lév.) S.H.Fu		Not Listed	Semisucculent perennial	Himalaya to China (SW. Sichuan, NW. Yunnan	Subalpine or subarctic	Wild Harvested	(53)
273	*Rhodiola coccinea*	Crassulaceae	*Rhodiola coccinea* (Royle) Boriss.		Not Listed	Perennial	Central Asia to SW. Siberia and W. & Central China	Subalpine or subarctic	Cultivated	(226)
266	*Rhodiola crenulata*	Crassulaceae	*Rhodiola crenulata* (Hook.f. & Thomson) H.Ohba		Not Listed	Geophyte	Himalaya to Qinghai and China (Sichuan, Yunnan)	Subalpine or subarctic	Wild Harvested	(53)
274	*Rhodiola gelida*	Crassulaceae	*Rhodiola gelida* Schrenk ex Fisch. & C.A.Mey.		Not Listed	Semisucculent Perennial	*Central Asia to Xinjiang*	Subtropical	Wild Harvested	(226)
275	*Rhodiola kirilowii*	Crassulaceae	*Rhodiola kirilowii* (Regel) Maxim.		Not Listed	Semisucculent Perennial	*Central Asia to Central China and Myanmar*	Subalpine or subarctic	Wild Harvested	(226)

276	*Rhodiola quadrifida*	Crassulaceae	*Rhodiola quadrifida* (Pall.) Fisch. & C.A.Mey.		Not Listed	Semisucculent Perennial	*Russia (Ural'skiy Chrebet) to Russian Far East and W. Himalaya*	Subalpine or subarctic	Wild Harvested	(226)
277	*Rhododendron anthopogonoides*	Ericaceae	*Rhododendron anthopogonoides* Maxim.		Not Listed	Shrub	Qinghai to China (NW. Sichuan, Guizhou)	Temperate	Wild Harvested	(103)
278	*Rhodomyrtus tomentosa*	Myrtaceae	*Rhodomyrtus tomentosa* (Aiton) Hassk.		LC	Shrub	Tropical & Subtropical Asia.	Wet tropical	Wild Harvested	(227)
279	*Rubia cordifolia*	Rubiaceae	*Rubia cordifolia* L.		Not Listed	Scambling Perenial	*Greece, Sudan to S. Africa, Asia*	Temperate	Wild Harvested	(228)
280	*Salvadora persica*	Salvadoraceae	*Salvadora persica* L.		LC	Shrub or Tree	Africa to Syria and Arabian Peninsula.	Dry tropical	Wild Harvested	(101)
281	*Salvia broussonetii*	Lamiaceae	*Salvia broussonetii* Bolle	*Salvia broussonetii* Benth.	Not Listed	Shrub or Subshrub	Canary Islands (Tenerife, Lanzarote?)	Subtropical	Wild Harvested	(45)
282	*Salvia candelabrum*	Lamiaceae	*Salvia candelabrum* Boiss.		LC	Shrub or Subshrub	S. Spain	Subtropical	Wild Harvested	(45)
283	*Salvia fruticosa*	Lamiaceae	*Salvia fruticosa* Mill.		LC	Shrub	Central & E. Medit	Subtropical	Cultivated and Wild Harvested	(51, 229)
284	*Salvia glutinosa*	Lamiaceae	*Salvia glutinosa* L.		Not Listed	Perennial	Europe to Iran	Subtropical	Wild Harvested	(45)
285	*Salvia herbanica*	Lamiaceae	*Salvia herbanica* A.Santos & M.Fernández		CR	Perennial	Canary Islands (Fuerteventura	Subtropical	Wild Harvested	(45)
286	*Salvia hydrangea*	Lamiaceae	*Salvia hydrangea* DC. ex Benth.		Not Listed	Subshrub	NE. Türkiye to Iran	Subtropical	Cultivated and Wild Harvested	(230)
287	*Salvia officinalis*	Lamiaceae	*Salvia officinalis* L.		LC	Subshrub	SW. Germany to S. Europe	Temperate	Cultivated	(76)

288	*Salvia officinalis subsp. Lavandulifolia*	Lamiaceae	*Salvia officinalis* subsp. *lavandulifolia* (Vahl) Gams		LC	Subshrub	Central & E. Spain	Temperate	Cultivatltivated and Wild Harvested	(229)
289	*Salvia Pomifera subsp. Pomifera*	Lamiaceae	*Salvia pomifera* subsp. *pomifera*		Not Listed	Shrub	S. Greece, Kriti.	Subtropical	Wild Harvested	(51)
290	*Sambucus adnata*	Adoxaceae	*Sambucus adnata* Wall. ex DC.		Not Listed	Shrub	Himalaya to China	Temperate	Wild Harvested	(231)
291	*Sambucus javanica*	Adoxaceae	*Sambucus javanica* Reinw. ex Blume		LC	Perennial	Afghanistan to Japan and Tropical Asia	Wet tropical	Wild Harvested	(231, 232)
292	*Sambucus nigra subsp. palmensis*	Caprifoliaceae	*Sambucus nigra* subsp. *palmensis* (Link) Bolli	*Sambucus palmensis* Link	EN	Shrub	Canary Islands (La Palma, Tenerife)	Subtropical biome	Wild Harvested	(45)
293	*Sambucus williamsii*	Adoxaceae	*Sambucus williamsii* Hance		LC	Shrub or Tree	S. Siberia to China and S. Korea, Japan	Temperate	Cultivated and Wild Harvested	(231)
294	*Santalum album*	Santalaceae	*Santalum album* L.		VU	Shrub or Tree	Jawa to N. Australia	Wet tropical	Wild Harvested	(44)
295	*Saposhnikovia divaricata*	Umbelliferae	*Saposhnikovia divaricata* (Turcz. ex Ledeb.) Schischk.		Not Listed	Perennial	S. Siberia to Korea and N. China	Temperate	Wild Harvested	(233)
296	*Saraca asoca*	Fabaceae	*Saraca asoca* (Roxb.) W.J.de Wilde		VU	Tree	*E. Pakistan to Myanmar and Sri Lanka*	Dry tropical	Wild Harvested	(234)
297	*Satyrium ciliatum*	Orchidaceae	*Satyrium ciliatum* Lindl.	*Satyrium nepalense* var. *ciliatum* (Lindl.) Hook.f.	Not listed	Tuberous geophyte	Himalaya to to China (NW. Hunan) and N. Myanmar	Temperate	Wild Harvested	(235)
298	*Satyrium nepalense*	Orchidaceae	*Satyrium nepalense* D.Don		Not Listed	Tuberous geophyte	Indian Subcontinent to E. Central China	Temperate	Wild Harvested	(141)

299	*Schefflera leucantha*	Araliaceae	*Schefflera leucantha* R.Vig.	*Heptapleurum leucanthum* (R.Vig.) Y.F.Deng	LC	Shrub	China (NW. Yunnan, SW. Guangxi) to N. Indo-China	Sub Tropical biome	Wild Harvested	(47)
300	*Schisandra sphenanthera*	Schisandraceae	*Schisandra sphenanthera* Rehder & E.H.Wilson		Not listed	Climber	Central & S. China	Temperate	Wild Harvested	(236)
301	*Scutellaria javanica*	Lamiaceae	*Scutellaria javanica* Jungh.		Not Listed	Perennial	Hainan, Indo-China to New Guinea	Wet tropical	Wild Harvested	(44)
302	*Senna didymobotrya*	Fabaceae	*Senna didymobotrya (Fresen.) H.S.Irwin & Barneby*		LC	Shrub or tree	Ethiopia to S. Tropical Africa	Tropical	Wild Harvested	(101)
303	*Sideritis barbellata*	Lamiaceae	*Sideritis barbellata* Mend.-Heuer		LC	Shrub or Subshrub	Canary Islands (Gran Canaria, Hierrero)	Subtropical	Wild Harvested	(45)
304	*Sideritis brevicaulis*	Lamiaceae	*Sideritis brevicaulis* Mend.-Heuer		Not Listed	Shrub or Subshrub	Canary Islands (NW. Tenerife)	Subtropical	Wild Harvested	(45)
305	*Sideritis cystosiphon*	Lamiaceae	*Sideritis cystosiphon* Svent.		CR	Subshrub	Canary Islands (W. Tenerife)	Subtropical	Wild Harvested	(45)
306	*Sideritis dasygnaphala*	Lamiaceae	*Sideritis dasygnaphala* (Webb & Berthel.) Clos		Not Listed	Shrub or Subshrub	Canary Islands (Gran Canaria)	Subtropical	Wild Harvested	(45)
307	*Sideritis dianica*	Lamiaceae	*Sideritis dianica* D.Rivera, Obón, De la Torre & A.Barber		Not Listed	Subshrub	E. Spain (Alicante)	Temperate	Wild Harvested	(45)
308	*Sideritis discolor*	Lamiaceae	*Sideritis discolor* Webb ex Bolle		CR	Subshrub	Canary Islands (N. Gran Canaria)	Temperate	Wild Harvested	(45)

309	*Sideritis eriocephala*	Lamiaceae	*Sideritis eriocephala* Marrero Rodr. ex Negrín & P.Pérez		Not Listed	Subshrub	Canary Islands (C. Tenerife)	Subtropical	Wild Harvested	(45)
310	*Sideritis ferrensis*	Lamiaceae	*Sideritis ferrensis* P.Pérez & Négrin		Not Listed	Subshrub	Canary Islands (El Hierro)	Subtropical	Wild Harvested	(45)
311	*Sideritis glauca*	Lamiaceae	*Sideritis glauca* Cav.		Not Listed	Subshrub	Spain (Alicante, NE. Murcia)	Temperate	Wild Harvested	(45)
312	*Sideritis grandiflora*	Lamiaceae	*Sideritis grandiflora* Salzm. ex Benth.		Not Listed	Subshrub	SW. Spain, N. Morocco	Subtropical	Wild Harvested	(45)
313	*Sideritis guayedrae*	Lamiaceae	*Sideritis guayedrae* Marrero Rodr.		Not Listed	Subshrub	Canary Islands (Gran Canaria	Subtropical	Wild Harvested	(45)
314	*Sideritis infernalis*	Lamiaceae	*Sideritis infernalis* Bolle		VU	Subshrub	Canary Islands (SW. Tenerife)	Subtropical	Wild Harvested	(45)
315	*Sideritis kuegleriana*	Lamiaceae	*Sideritis kuegleriana* Bornm.		Not Listed	Subshrub	Canary Islands (N. Tenerife	Subtropical	Wild Harvested	(45)
316	*Sideritis lotsyi*	Lamiaceae	*Sideritis lotsyi* (Pit.) Bornm.		Not Listed	Shrub or subshrub	Canary Islands (Tenerife, La Gomera)	Subtropical	Wild Harvested	(45)

317	*Sideritis marmorea*	Lamiaceae	*Sideritis marmorea* Bolle		CR	Subshrub	Canary Islands (E. La Gomera)	Subtropical	Wild Harvested	(45)
318	*Sideritis nervosa*	Lamiaceae	*Sideritis nervosa* (Christ) Linding.		Not Listed	Subshrub	Canary Islands (NW. Tenerife)	Subtropical	Wild Harvested	(45)
319	*Sideritis nutans*	Lamiaceae	*Sideritis nutans* Svent.		Not Listed	Shrub	Canary Islands (W. La Gomera)	Subtropical	Wild Harvested	(45)
320	*Sideritis oroteneriffae*	Lamiaceae	*Sideritis oroteneriffae* Négrin & P.Pérez		Not Listed	Shrub or Subshrub	Canary Islands (Tenerife)	Subtropical	Wild Harvested	(45)
321	*Sideritis pumila*	Lamiaceae	*Sideritis pumila* (Christ) Mend.-Heuer		Not Listed	Subshrub	Canary Islands (Lanzarote, S. Fuerteventura).	Subtropical	Wild Harvested	(45)
322	*Sideritis reverchonii*	Lamiaceae	*Sideritis reverchonii* Willk.	*Sideritis tragoriganum* subsp. *reverchonii* (Willk.) R.Roselló,	EN	Subshrub	Spain (Cádiz, Malagá).	Subtropical	Wild Harvested	(45)
323	*Sideritis serrata*	Lamiaceae	*Sideritis serrata* Lag.		CR	Subshrub	SE. Spain (Albacete, Tobarra)	Temperate	Wild Harvested	(45)

324	*Sideritis spicata*	Lamiaceae	*Sideritis spicata* (Pit.) Marrero Rodr.	*Sideritis cretica* subsp. *spicata* (Pit.) Negrín & P.Pérez	Not Listed	Subshrub	Canary Islands (N. La Gomera)	Subtropical	Wild Harvested	(45)
325	*Sideritis stachydioides*	Lamiaceae	*Sideritis stachydioides* Willk.		Not Listed	Subshrub	SE. Spain (Vélez Rubio, Vélez Blanco)	Subtropical	Wild Harvested	(45)
326	*Sideritis sventenii*	Lamiaceae	*Sideritis sventenii* (G.Kunkel) Mend.-Heuer		Not Listed	Subshrub	Canary Islands (S. Central Gran Canaria).	Subtropical	Wild Harvested	(45)
327	*Solonum incanum*	Solanaceae	*Solanum incanum* L.		LC	Shrub or subshrub	Africa, Arabian Peninsula, Iran to NW. India	Tropical	Cultivated and Wild Harvested	(101)
328	*Sophora alopecuroides*	Fabaceae	*Sophora alopecuroides* L.		Not Listed	Perennial	E. Europe to Temp. Asia	Subtropical	Wild Harvested	(237)
329	*Stachys inflata*	Lamiaceae	*Stachys inflata* Benth.		Not Listed	Subshrub	NE. Türkiye to Iran	Temperate	Wild Harvested	(238)
330	*Stryphnodendron pulcherrimum*	Fabaceae	*Stryphnodendron pulcherrimum* (Willd.) Hochr.		LC	Tree	S. Tropical America.	Wet tropical	Wild Harvested	(239)

331	*Styrax sumatrana*	Styracaceae	*Styrax sumatranus* J.J.Sm.	*Styrax parallelone urus* Perkins	Not Listed	Tree	Thailand to Sumatera	Wet tropical	Wild Harvested	(240)
332	*Swertia petiolata*	Gentianaceae	*Swertia petiolata* D.Don		Not Listed	Perennial	*E. Afghanistan to W. & Central Himalaya*	Subalpine or subarctic	Wild Harvested	(241)
333	*Tagetes lucida*	Asteraceae	*Tagetes lucida* Cav.		Not Listed	Perennial	Mexico to Honduras	Subtropical	Cultivated	(242)
334	*Taxus baccata*	Taxaceae	*Taxus baccata* L.		Not Listed	Tree	Azores, Europe to N. Iran, NW. Africa.	Temperate	Wild Harvested	(243)
335	*Taxus wallichiana*	Taxaceae	*Taxus wallichiana* Zucc.		EN	Tree	Central Himalaya to China (NW. Yunnan), SE. Vietnam, Sumatera, Philippines, Sulawesi	Temperate	Wild Harvested	(244)
336	*Tecomella undulata*	Bignoniaceae	*Tecomella undulata* (Sm.) Seem.		EN	Tree	*Oman, SW. Iran to NW. India.*	Dry surbland	Wild Harvested	(245)
337	*Terminalia chebula*	Combretaceae	*Terminalia chebula* Retz.		LC	Tree	Indian Subcontinent to China (W. Yunnan) and Indo-China	Wet tropical	Wild Harvested	(246)

338	*Tetrastigma hemsleyanum*	Vitaceae	*Tetrastigma hemsleyanum* Diels & Gilg		Not Listed	Liana	*Tibet to S. China, Taiwan*	Temperate	Wild Harvested	(247)
339	*Thesium chinense*	Santalaceae	*Thesium chinense* Turcz.		Not Listed	Perennial	SE. Siberia to China and Temp. E. Asia	Temperate	Wild Harvested	(58)
340	*Thymbra capitata*	Lamiaceae	*Thymbra capitata* (L.) Cav.		LC	Subshrub	Mediterania	Subtropical	Cultivated and Wild Harvested	(51)
341	*Thymus albicans*	Lamiaceae	*Thymus albicans* Hoffmanns. & Link		VU	Subshrub	S. Portugal to SW. Spain.	Subtropical	Wild Harvested	(45)
342	*Thymus carnosus*	Lamiaceae	*Thymus carnosus* Boiss.		NT	Subshrub	SW. & S. Portugal to SW	Subtropical	Wild Harvested	(45)
343	*Thymus daenensis*	Lamiaceae	*Thymus daenensis* Čelak		Not Listed	Subshrub	*N. Iraq to Iran*	Temperate	Cultivated and Wild Harvested	(248)
344	*Thymus daenensis subsp. daenensis*	Lamiaceae	*Thymus daenensis* subsp. *daenensis*		Not Listed	Subshrub	*N. Iraq to Iran*	Temperate	Cultivated and Wild Harvested	(249)
345	*Thymus daenensis subsp. lancifolius*	Lamiaceae	*Thymus daenensis* subsp. *lancifolius* (Čelak.) Jalas		Not Listed	Subshrub	*N. Iraq to Iran*	Temperate	Cultivated and Wild Harvested	(249)

346	*Thymus kotschyanus*	Lamiaceae	*Thymus kotschyanus* Boiss. & Hohen.		Not Listed	Subshrub	S. & E. Türkiye to Iran	Temperate	Wild Harvested	(248)
347	*Thymus linearis*	Lamiaceae	*Thymus linearis* Benth.		Not Listed	Subshrub	N. Iran to Xinjiang and Himalaya	Temperate	Wild Harvested	(118)
348	*Thymus transcaucasicus*	Lamiaceae	*Thymus transcaucasicus* Ronniger		Not Listed	Subshrub	NE. Türkiye to N. Iran	Temperate	Wild Harvested	(250)
349	*Thymus vulgaris*	Lamiaceae	*Thymus vulgaris* L.		LC	Subshrub	SW. Europe, SE. Italy	Temperate	Cultivated and Wild Harvested	(74, 142, 251)
350	*Tilia amurensis*	Tiliaceae	*Tilia amurensis* Rupr.		LC	Tree	Russian Far East to Korea	Temperate	Wild Harvested	(252)
351	*Tinospora craveniana*	Menispermaceae	*Tinospora craveniana* S.Y.Hu	*Paratinospora sagittata* (Oliv.) Wei Wang	Not Listed	Climbing shrub	China to N. Vietnam	Temperate	Wild Harvested	(253)
352	*Tinospora sinensis*	Menispermaceae	*Tinospora sinensis* (Lour.) Merr.		Not Listed	Liana	ndian Subcontinent to China (S. Yunnan to S. Guangdong) and Indo-China	Wet tropical	Wild Harvested	(253)

353	*Tinospora yunnanensis*	Menispermaceae	*Tinospora yunnanensis* S.Y.Hu	*Paratinospora sagittata* (Oliv.) Wei Wang	Not Listed	Climbing shrub	China to N. Vietnam	Temperate	Wild Harvested	(253)
354	*Tulbaghia violacea*	Amaryllidaceae	*Tulbaghia violacea* Harv.		Not Listed	Bulbous geophyte	Cape Prov. to KwaZulu-Natal.	Subtropical	Wild Harvested	(71)
355	*Tylophora hirsuta*	Apocynaceae	*Tylophora hirsuta* (Wall.) Wight	*Vincetoxicum hirsutum* (Wall.) Kuntze	Not Listed	Liana	Indian Subcontinent to Taiwan and Peninsula Malaysia, W. Jawa	Subtropical	Wild Harvested	(210)
356	*Tymus fedschenkoi*	Lamiaceae	*Thymus fedtschenkoi* Ronniger		Not Listed	Subshrub	E. Türkiye to Iran.	Temperate	Wild Harvested	(250)
357	*Tymus pubescens*	Lamiaceae	*Thymus pubescens* Boiss. & Kotschy ex Čelak.		Not Listed	Subshrub	E. Türkiye to Iran	Temperate	Wild Harvested	(250)
358	*Urtica dioica*	Urticaceae	*Urtica dioica* L.		LC	Perennial or rhizomatous geophyte	Europe to Siberia and W. China, NW. Africa.	Temperate	Wild Harvested	(118)
359	*Valeriana carnosa*	Caprifoliaceae	*Valeriana carnosa* Sm.		Not Listed	Perennial	Central & S. Chile to W. & S. Argentina	Temperate	Wild Harvested	(254)

360	*Valeriana jatamansi*	Valerianaceae	*Valeriana jatamansi* Jones ex Roxb.		Not Listed	Perennial	E. Afghanistan to Central & E. Central China and N. Indo-China	Temperate	Wild Harvested	(106)
361	*Valeriana wallichii*	Valerianaceae	*Valeriana wallichii* DC.	*Valeriana jatamansi* Jones ex Roxb.	Not Listed	Perennial	E. Afghanistan to Central & E. Central China and N. Indo-China.	Temperate	Wild Harvested	(255)
362	*Vincetoxicum arnottianum*	Apocynaceae	*Vincetoxicum arnottianum* (Wight) Wight		Not Listed	Perennial	N. Pakistan to W. Himalaya	Temperate	Wild Harvested	(210)
363	*Warburgia salutaris*	Canellaceae	*Warburgia salutaris* (G.Bertol.) Chiov.		VU	Shrub or Tree	Zimbabwe to KwaZulu-Natal	Dry tropical	Wild Harvested	(256)
364	*Zanthoxylum armatum*	Rutaceae	*Zanthoxylum armatum* (Thunb.) Druce	*Zanthoxylum capense* (Thunb.) Harv.	LC	Shrub or Tree	Indian Subcontinent to Temp. E. Asia and Malesia.	Temperate	Cultivated and Wild Harvested	(257)
365	*Zanthoxylum nitidum*	Rutaceae	*Zanthoxylum nitidum* (Roxb.) DC.		LC	Shrub	Asia to NE. Australia	Wet tropical	Cultivated and Wild Harvested	(258)
366	*Zingiber striolatum*	Zingiberaceae	*Zingiber striolatum* Diels		Not Listed	Perennial	S. China	Subtropical	Wild Harvested	(259)
367	*Ziziphus spinosa*	Rhamnaceae	*Ziziphus spinosa* St.-Lag.	*Paliurus spina-christi* Mill.	Not Listed	Shrub or tree	Medit. to Central Asia and NW. Aghanistan	Subtropical	Wild Harvested	(260)

Note: CR : Critical Endangered
EN : Endangered
VU : Vulnerable
NT : Near Threatened
DD : Data Deficient

REFERENCES

1. Kwak S-S, Choi M-S, Park Y-G, Yoo J-S, Liu J-R. Taxol content in the seeds of Taxus spp. Phytochemistry. 1995;40(1):29-32.
2. Chen M, Yan T, Shen Q, Lu X, Pan Q, Huang Y, et al. Glandular Trichome Specific 1 promotes artemisinin biosynthesis in Artemisia annua. New Phytologist. 2017;214(1):304-16.
3. Yuan H, Ma Q, Ye L, Piao G. The traditional medicine and modern medicine from natural products. Molecules. 2016;21(5):559.
4. Lee M. Colchicum Autumnale and the Gout. Naked Ladies and Portly Gentlemen. Journal of the Royal College of Physicians of Edinburgh. 1999;29(1):65-70.
5. Balunas MJ, Kinghorn AD. Drug discovery from medicinal plants. Life sciences. 2005;78(5):431-41.
6. Morton EM, Rafferty NE. Plant–pollinator interactions under climate change: The use of spatial and temporal transplants. Applications in plant sciences. 2017;5(6):1600133.
7. Dybzinski R, Fargione JE, Zak DR, Fornara D, Tilman D. Soil fertility increases with plant species diversity in a long-term biodiversity experiment. Oecologia. 2008;158:85-93.
8. Tomlinson TR, Akerele O. Medicinal plants: their role in health and biodiversity: University of Pennsylvania press; 2015.
9. Kala CP. Medicinal and aromatic plants of Uttarakhand: An important ecosystem service for human well-being. Ecosystem Services and Its Mainstreaming in Development Planning Process; Bishen Singh Mahendra Pal Singh Dehradun. 2015:145-54.
10. Schippmann U, Leaman DJ, Cunningham AB. Impact of cultivation and gathering of medicinal plants on biodiversity: global trends and issues. FAO; 2002.
11. Heinrich M, Ankli A, Frei B, Weimann C, Sticher O. Medicinal plants in Mexico: Healers' consensus and cultural importance. Social science & medicine. 1998;47(11):1859-71.
12. Upa MSMP, Bessi MIT, Korassa YB, Indrawati MI. Ethnopharmacology Study of Traditional Herbs as Anti-dandruff and Anti-baldness in Amarasi District, Kupang Regency. Jurnal Farmasi Galenika (Galenika Journal of Pharmacy)(e-Journal). 2023;9(2):180-8.
13. Ambu G, Chaudhary RP, Mariotti M, Cornara L. Traditional uses of medicinal plants by ethnic people in the Kavrepalanchok district, Central Nepal. Plants. 2020;9(6):759.
14. WHO. WHO traditional medicine strategy: 2014-2023. 2013.
15. Heinrich M, Teoh HL. Galanthamine from snowdrop—the development of a modern drug against Alzheimer's disease from local Caucasian knowledge. Journal of ethnopharmacology. 2004;92(2-3):147-62.
16. Lin HQ, Gong AG, Wang HY, Duan R, Dong TT, Zhao KJ, et al. Danggui Buxue tang (Astragali Radix and Angelicae Sinensis Radix) for menopausal symptoms: a review. Journal of Ethnopharmacology. 2017;199:205-10.
17. Nainwal P, Singh N. Sustainable Use of High Altitude Medicinal and Aromatic Plants for Socio-Economic Development in Uttarakhand: A Review. International Journal of Pharmaceutical Science and Research. 2020:4238-43.
18. Asigbaase M, Adusu D, Anaba L, Abugre S, Kang-Milung S, Acheamfour SA, et al. Conservation and economic benefits of medicinal plants: Insights from forest-fringe communities of Southwestern Ghana. Trees, Forests and People. 2023;14:100462.
19. Moisiienko V, Nazarchyk O. Yield of chamomile medicinal depending on sowing date and fertilizing in terms of climate change. Scientific Horizons. 2019;2(22):3-12.

20. Mansinhos I, Gonçalves S, Romano A. How climate change-related abiotic factors affect the production of industrial valuable compounds in Lamiaceae plant species: a review. Frontiers in Plant Science. 2024;15:1370810.
21. Laftouhi A, Eloutassi N, Ech-Chihbi E, Rais Z, Taleb A, Assouguem A, et al. Impact of Climate Change on the Chemical Compositions and Antioxidant Activity of Mentha pulegium L. ACS omega. 2023;8(49):46598-607.
22. Groner VP, Nicholas O, Mabhaudhi T, Slotow R, Akçakaya HR, Mace GM, et al. Climate change, land cover change, and overharvesting threaten a widely used medicinal plant in S outh A frica. Ecological Applications. 2022;32(4):e2545.
23. Mansour-Gueddes SB, Saidana-Naija D, Bchir A, Braham M. Climate change effects on phytochemical compounds and antioxidant activity of Olea europaea L. Notulae Botanicae Horti Agrobotanici Cluj-Napoca. 2020;48(1):436-55.
24. Omidi H, Shams H, Sahandi MS, Rajabian T. Balangu (Lallemantia sp.) growth and physiology under field drought conditions affecting plant medicinal content. Plant Physiology and Biochemistry. 2018;130:641-6.
25. Tricco AC, Lillie E, Zarin W, O'Brien KK, Colquhoun H, Levac D, et al. PRISMA extension for scoping reviews (PRISMA-ScR): checklist and explanation. Annals of internal medicine. 2018;169(7):467-73.
26. McGowan J, Straus S, Moher D, Langlois EV, O'Brien KK, Horsley T, et al. Reporting scoping reviews-PRISMA ScR extension. J Clin Epidemiol. 2020;123(177):e9.
27. Wang W-Y, Zhou H, Wang Y-F, Sang B-S, Liu L. Current policies and measures on the development of traditional Chinese medicine in China. Pharmacological research. 2021;163:105187.
28. Cunningham A, Brinckmann J, Pei S-J, Luo P, Schippmann U, Long X, et al. High altitude species, high profits: can the trade in wild harvested Fritillaria cirrhosa (Liliaceae) be sustained? Journal of ethnopharmacology. 2018;223:142-51.
29. Zhao Q, Li R, Gao Y, Yao Q, Guo X, Wang W. Modeling impacts of climate change on the geographic distribution of medicinal plant Fritillaria cirrhosa D. Don. Plant Biosystems-An International Journal Dealing with all Aspects of Plant Biology. 2018;152(3):349-55.
30. Zhao Z, Guo P, Brand E. The formation of daodi medicinal materials. Journal of ethnopharmacology. 2012;140(3):476-81.
31. Li J, Deng C, Duan G, Wang Z, Zhang Y, Fan G. Potentially suitable habitats of Daodi goji berry in China under climate change. Frontiers in Plant Science. 2024;14:1279019.
32. Singh UM, Vijayta Gupta VG, Rao V, Sengar RS, Yadav M. A review on biological activities and conservation of endangered medicinal herb Nardostachys jatamansi. International Journal of Medicinal and Aromatic Plants 2013;3:113-24.
33. Arora R, Arora C, editors. Hypotensive and tranquillizing activity of jatamansone (valeranone) a sesquiterpene from Nardostachys jatamansi DC. Pharmacology of Oriental Plants: Proceedings of the First International Pharmacological Meeting, Stockholm, 22-25 August, 1961; 2016: Elsevier.
34. Bhagwan SC, Gupta VS, Deshmukh VV, Sardeshmukh SS, Sardeshmukh SP. Standardization and preliminary characterization of an ayurvedic stress-relieving head massage oil of Nardostachys jatamansi DC. Journal of Ayurveda and Integrative Medicine. 2024;15(2):100900.
35. Hashim YZH-Y, Kerr PG, Abbas P, Salleh HM. Aquilaria spp.(agarwood) as source of health beneficial compounds: A review of traditional use, phytochemistry and pharmacology. Journal of Ethnopharmacology. 2016;189:331-60.

36. Mykhailenko O, Jalil B, McGaw LJ, Echeverría J, Takubessi M, Heinrich M. Climate change and the sustainable use of medicinal plants: a call for “new” research strategies. Frontiers in Pharmacology. 2025;15:1496792.
37. Indrioko S, Ratnaningrum YW. Habitat loss caused clonality, genetic diversity reduction and reproductive failure in Santalum album (Santalaceae), an endangered endemic species of Indonesia. Procedia Environmental Sciences. 2015;28:657-64.
38. Ratnaningrum YW, Indrioko S. Response of flowering and seed production of sandalwood (Santalum album linn., Santalaceae) to climate changes. Procedia Environmental Sciences. 2015;28:665-75.
39. Kumar R, Anjum N, Tripathi Y. Phytochemistry and pharmacology of Santalum album L.: a review. World Journal of Pharmaceutical Research. 2015;4(10):1842-76.
40. Saneja A, Kaushik P, Kaushik D, Kumar S, Kumar D. Antioxidant, analgesic and anti-inflammatory activities of Santalum album Linn. Planta Medica. 2009;75(04):P-102.
41. Kumar P, Rana V, Singh AN. Angelica glauca Edgew.–a comprehensive review. Journal of Applied Research on Medicinal and Aromatic Plants. 2022;31:100397.
42. Khaleeq-Uz-Zaman JB, Shafi, Mohammad Munir, Iqbal Phtyto-chemical composition, antimicrobial and phyto-toxic activity of angelica glauca (apiaceae). Pak J Bot. 2018;50(5):1893-8.
43. Mahmoodi S, Heydari M, Ahmadi K, Khwarahm NR, Karami O, Almasieh K, et al. The current and future potential geographical distribution of Nepeta crispa Willd., an endemic, rare and threatened aromatic plant of Iran: Implications for ecological conservation and restoration. Ecological Indicators. 2022;137:108752.
44. Cahyaningsih R, Phillips J, Magos Brehm J, Gaisberger H, Maxted N. Climate change impact on medicinal plants in Indonesia. Global Ecol Conserv 30: e01752. 2021.
45. Munt DD, Muñoz-Rodríguez P, Marques I, Saiz JCM. Effects of climate change on threatened Spanish medicinal and aromatic species: predicting future trends and defining conservation guidelines. Israel Journal of Plant Sciences. 2016;63(4):309-19.
46. Kumar D, Singh M, Sharma S. Fate of important medicinal plants in the eastern Himalaya in changing climate scenarios: a case of Panax pseudoginseng Wall. Applied Ecology & Environmental Research. 2019;17(6).
47. Hao Y, Dong P, Wang L, Ke X, Hao X, He G, et al. Predicting the Potential Distribution of Hypericum perforatum under Climate Change Scenarios Using a Maximum Entropy Model. Biology. 2024;13(6):452.
48. Souther S, McGraw JB. Synergistic effects of climate change and harvest on extinction risk of American ginseng. Ecological Applications. 2014;24(6):1463-77.
49. Rajpoot R, Adhikari D, Verma S, Saikia P, Kumar A, Grant KR, et al. Climate models predict a divergent future for the medicinal tree Boswellia serrata Roxb. in India. Global Ecology and Conservation. 2020;23:e01040.
50. Liu M, Li L, Wang S, Xiao S, Mi J. Forecasting the future suitable growth areas and constructing ecological corridors for the vulnerable species Ephedra sinica in China. Journal for Nature Conservation. 2023;73:126401.
51. Bariotakis M, Georgescu L, Laina D, Koufaki M, Souma M, Douklias S, et al. Climate Change Dependence in Ex Situ Conservation of Wild Medicinal Plants in Crete, Greece. Biology. 2023;12(10):1327.

52. Qiu L, Fu Q-L, Jacquemyn H, Burgess KS, Cheng J-J, Mo Z-Q, et al. Contrasting range changes of Bergenia (Saxifragaceae) species under future climate change in the Himalaya and Hengduan Mountains Region. Theoretical and Applied Climatology. 2024;155(3):1927-39.
53. Wang W, Yang T, Jin L, Jiang J. Vulnerability of two Rhodiola species under climate change in the future. Biodiversity Science. 2021;29(12):1620.
54. Pan C, Chen S, Chen Z, Li Y, Liu Y, Zhang Z, et al. Assessing the geographical distribution of 76 Dendrobium species and impacts of climate change on their potential suitable distribution area in China. Environmental Science and Pollution Research. 2022;29(14):20571-92.
55. Jung JB, Park GE, Kim HJ, Huh JH, Um Y. Predicting the habitat suitability for Angelica gigas medicinal herb using an ensemble species distribution model. Forests. 2023;14(3):592.
56. Singh L, Kanwar N, Bhatt ID, Nandi SK, Bisht AK. Predicting the potential distribution of Dactylorhiza hatagirea (D. Don) Soo-an important medicinal orchid in the West Himalaya, under multiple climate change scenarios. PLoS One. 2022;17(6):e0269673.
57. Vihotogbé R, Idohou R, Vianou A, Spies P, Salako V, Assogbadjo A, et al. Abundance and effects of climate change on geographical distribution of Mondia whitei (Hook. f.) Skeels (Apocynaceae) in the Dahomey Gap (West Africa). African Journal of Ecology. 2021;59(4):924-33.
58. Zhang B, Chen B, Zhou X, Zou H, Duan D, Zhang X, et al. Distribution and protection of Thesium chinense Turcz. under climate and land use change. Scientific Reports. 2024;14(1):6475.
59. Ngaruiya GW. Reweaving stakeholder networks: Promoting climate mitigation and Maasai culture using medicinal plants in Kenya. Ecosystem Services. 2015;15:103-12.
60. Lv G, Li Z, Zhao Z, Liu H, Li L, Li M. The factors affecting the development of medicinal plants from a value chain perspective. Planta. 2024;259(5):108.
61. Singh PA, Dash S, Choudhury A, Bajwa N. Factors affecting long-term availability of medicinal plants in India. Journal of Crop Science and Biotechnology. 2024;27(2):145-73.
62. Ngarega BK, Chaibva P, Masocha VF, Saina JK, Khine PK, Schneider H. Application of MaxEnt modeling to evaluate the climate change effects on the geographic distribution of Lippia javanica (Burm. f.) Spreng in Africa. Environmental Monitoring and Assessment. 2024;196(1):62.
63. Brower V. Back to nature: extinction of medicinal plants threatens drug discovery. Oxford University Press; 2008.
64. Mendelsohn R, Balick MJ. The value of undiscovered pharmaceuticals in tropical forests. Economic botany. 1995;49:223-8.
65. Newman DJ, Cragg GM, Snader KM. Natural products as sources of new drugs over the period 1981− 2002. Journal of natural products. 2003;66(7):1022-37.
66. Brinckmann JA. Geographical indications for medicinal plants: globalization, climate change, quality and market implications for geo-authentic botanicals. World Journal of Traditional Chinese Medicine. 2015;1(1):16-23.
67. Dias MC, Pinto DC, Costa M, Araújo M, Santos C, Silva AM. Phytochemical and antioxidant profile of the medicinal plant Melia azedarach subjected to water deficit conditions. International Journal of Molecular Sciences. 2022;23(21):13611.
68. Cáceres-Cevallos GJ, Albacete-Moreno AA, Ferreres F, Gil-Izquierdo Á, Jordán MJ. Evaluation of the physiological parameters in Lavandula latifolia Medik. under water deficit for preselection of elite drought-resistant plants. Industrial Crops and Products. 2023;199:116742.
69. Dobhal P, Purohit VK, Chandra S, Rawat S, Prasad P, Bhandari U, et al. Climate-induced changes in essential oil production and terpene composition in alpine aromatic plants. Plant Stress. 2024;12:100445.

70. Chang J-d, Mantri N, Sun B, Jiang L, Chen P, Jiang B, et al. Effects of elevated CO2 and temperature on Gynostemma pentaphyllum physiology and bioactive compounds. Journal of Plant Physiology. 2016;196:41-52.
71. Netshiluvhi TR, Eloff JN. Temperature and water stresses on antioxidant activity of selected medicinal plants have implications for sustainable use and global warming. South African Journal of Botany. 2024;170:177-80.
72. Medda S, Fadda A, Mulas M. Climate variables of the sites of origin and genotype influence on phenolic compounds accumulation in cultivars of Myrtus communis L. Horticulturae. 2022;8(10):928.
73. Kumar S, Yadav A, Yadav M, Yadav JP. Effect of climate change on phytochemical diversity, total phenolic content and in vitro antioxidant activity of Aloe vera (L.) Burm. f. BMC research notes. 2017;10:1-12.
74. Kaur T, Bhat R, Vyas D. Effect of contrasting climates on antioxidant and bioactive constituents in five medicinal herbs in Western Himalayas. Journal of Mountain Science. 2016;13:484-92.
75. Shen T, Yu H, Wang Y-Z. Assessing the impacts of climate change and habitat suitability on the distribution and quality of medicinal plant using multiple information integration: Take Gentiana rigescens as an example. Ecological Indicators. 2021;123:107376.
76. Copolovici L, Copolovici DM, Moisa C, Lupitu A. Antagonist temperature variation affects the photosynthetic parameters and secondary metabolites of Ocimum basilicum L. and Salvia officinalis L. Plants. 2022;11(14):1806.
77. Trenberth KE, Dai A, Van Der Schrier G, Jones PD, Barichivich J, Briffa KR, et al. Global warming and changes in drought. Nature Climate Change. 2014;4(1):17-22.
78. Tan U, Gören HK. Comprehensive evaluation of drought stress on medicinal plants: a meta-analysis. PeerJ. 2024;12:e17801.
79. Ai Q, Dai A, Han M, Yang L, Liu C. Moderate Drought Stress Interferes with the Physiological State and Promotes the Accumulation of Isoflavone in Reproductive Iris domestica Rhizomes. Agronomy. 2024;14(8):1730.
80. Li T, Tiiva P, Rinnan Å, Julkunen-Tiitto R, Michelsen A, Rinnan R. Long-term effects of elevated CO2, nighttime warming and drought on plant secondary metabolites in a temperate heath ecosystem. Annals of Botany. 2020;125(7):1065-75.
81. Jamloki A, Bhattacharyya M, Nautiyal M, Patni B. Elucidating the relevance of high temperature and elevated CO2 in plant secondary metabolites (PSMs) production. Heliyon. 2021;7(8).
82. Li Z-G. Mechanisms of plant adaptation and tolerance to heat stress. Plant ecophysiology and adaptation under climate change: mechanisms and perspectives II: mechanisms of adaptation and stress amelioration2020. p. 39-59.
83. Ashraf MV, Khan S, Misri S, Gaira KS, Rawat S, Rawat B, et al. High-altitude medicinal plants as promising source of phytochemical antioxidants to combat lifestyle-associated oxidative stress-induced disorders. Pharmaceuticals. 2024;17(8):975.
84. Prinsloo G, Nogemane N. The effects of season and water availability on chemical composition, secondary metabolites and biological activity in plants. Phytochemistry Reviews. 2018;17(4):889-902.
85. Midzi J, Jeffery DW, Baumann U, Rogiers S, Tyerman SD, Pagay V. Stress-induced volatile emissions and signalling in inter-plant communication. Plants. 2022;11(19):2566.

86. Jalil B, Heinrich M. Pharmaceutical quality of herbal medicinal products and dietary supplements–a case study with oral solid formulations containing Lavandula species. European Journal of Pharmaceutical Sciences. 2025;208:107042.
87. Kulak M. Recurrent drought stress effects on essential oil profile of Lamiaceae plants: An approach regarding stress memory. Industrial Crops and Products. 2020;154:112695.
88. Sarmoum R, Haid S, Biche M, Djazouli Z, Zebib B, Merah O. Effect of salinity and water stress on the essential oil components of rosemary (Rosmarinus officinalis L.). Agronomy. 2019;9(5):214.
89. Maikhuri R, Phondani P, Dhyani D, Rawat L, Jha N, Kandari L. Assessment of climate change impacts and its implications on medicinal plants-based traditional healthcare system in Central Himalaya, India. Iranian Journal of Science and Technology, Transactions A: Science. 2018;42:1827-35.
90. Tangjitman K, Trisonthi C, Wongsawad C, Jitaree S. Potential impact of climatic change on medicinal plants used in the Karen women's health care in northern Thailand. Songklanakarin Journal of Science & Technology. 2015;37(3).
91. Mon AM, Hein PP, Zaw M, Kyaw MT, Yang Y, Yang X, et al. Ethnobotanical surveys reveal the crucial role of medicinal plants in the primary healthcare system of the Shan people in Myanmar. Journal of Ethnopharmacology. 2024;327:117875.
92. Rahman MH, Roy B, Chowdhury GM, Hasan A, Saimun MSR. Medicinal plant sources and traditional healthcare practices of forest-dependent communities in and around Chunati Wildlife Sanctuary in southeastern Bangladesh. Environmental Sustainability. 2022;5(2):207-41.
93. Cunningham AB. Applied ethnobotany: people, wild plant use and conservation: Routledge; London. 2014.
94. Palit P, Mandal SC. Climate Change, Geographical Location, and Other Allied Triggering Factors Modulate the Standardization and Characterization of Traditional Medicinal Plants: A Challenge and Prospect for Phyto-drug Development. In: Mandal SC, Chakraborty R, Sen S, editors. Evidence Based Validation of Traditional Medicines: A comprehensive Approach. Singapore: Springer Singapore; 2021. p. 359-69.
95. Fajinmi OO, Olarewaju OO, Van Staden J. Propagation of medicinal plants for sustainable livelihoods, economic development, and biodiversity conservation in South Africa. Plants. 2023;12(5):1174.
96. Giliba RA, Yengoh GT. Predicting suitable habitats of the African cherry (Prunus Africana) under climate change in Tanzania. Atmosphere. 2020;11(9):988.
97. Groner VP, Nicholas O, Mabhaudhi T, Slotow R, Akçakaya HR, Mace GM, et al. Climate change, land cover change, and overharvesting threaten a widely used medicinal plant in South Africa. Ecological Applications. 2022;32(4).
98. Feng L, Sun J, Shi Y, Wang G, Wang T. Predicting suitable habitats of Camptotheca acuminata considering both climatic and soil variables. Forests. 2020;11(8):891.
99. Peralta PA, Nores MJ, Bach HG, Robbiati FO. Facing climate change: Range dynamics and chromosome diversity in Hedeoma multiflora Benth., a South American aromatic-medicinal plant at risk. Flora. 2024;315:152519.
100. Zou H, Zhang B, Chen B, Duan D, Zhou X, Chen J, et al. A multi-dimensional" climate-land-quality" approach to conservation planning for medicinal plants: Take Gentiana scabra Bunge in China as an example. Industrial Crops and Products. 2024;211:118222.
101. Gafna DJ, Obando JA, Kalwij JM, Dolos K, Schmidtlein S. Climate change impacts on the availability of anti-malarial plants in Kenya. Climate Change Ecology. 2023;5:100070.

102. Noedoost F, Behroozian M, Karami S, Joharchi MR. Potential impacts of climate change on the geographic distribution of Achillea eriophora DC., a medicinal species endemic to Iran in southwestern Asia. Ecology and Evolution. 2024;14(4):e11241.
103. Yang L, Zhu X, Song W, Shi X, Huang X. Predicting the potential distribution of 12 threatened medicinal plants on the Qinghai-Tibet plateau, with a maximum entropy model. Ecology and Evolution. 2024;14(2):e11042.
104. Dad JM, Rashid I. Differential responses of Kashmir Himalayan threatened medicinal plants to anticipated climate change. Environmental Conservation. 2022;49(1):33-41.
105. Kunwar RM, Thapa-Magar KB, Subedi SC, Kutal DH, Baral B, Joshi NR, et al. Distribution of important medicinal plant species in Nepal under past, present, and future climatic conditions. Ecological Indicators. 2023;146:109879.
106. Rana SK, Rana HK, Ranjitkar S, Ghimire SK, Gurmachhan CM, O'Neill AR, et al. Climate-change threats to distribution, habitats, sustainability and conservation of highly traded medicinal and aromatic plants in Nepal. Ecological Indicators. 2020;115:106435.
107. Wang Y, Zhao R, Zhou X, Zhang X, Zhao G, Zhang F. Prediction of potential distribution areas and priority protected areas of Agastache rugosa based on Maxent model and Marxan model. Frontiers in Plant Science. 2023;14:1200796.
108. Zhang J-M, Song M-L, Li Z-J, Peng X-Y, Su S, Li B, et al. Effects of climate change on the distribution of Akebia quinata. Frontiers in Ecology and Evolution. 2021;9:752682.
109. Zhang JM, Peng XY, Song ML, Li ZJ, Xu XQ, Wang W. Effects of climate change on the distribution of wild Akebia trifoliata. Ecology and evolution. 2022;12(3):e8714.
110. Tshabalala T, Mutanga O, Abdel-Rahman EM. Predicting the geographical distribution shift of medicinal plants in South Africa due to climate change. Conservation. 2022;2(4):694-708.
111. Asase A, Peterson AT. Predicted impacts of global climate change on the geographic distribution of an invaluable African medicinal plant resource, Alstonia boonei De Wild. Journal of Applied Research on Medicinal and Aromatic Plants. 2019;14:100206.
112. Silva JLS, Cruz-Neto O, Tabarelli M, Albuquerque UP, Lopes AV. Climate change will likely threaten areas of suitable habitats for the most relevant medicinal plants native to the Caatinga dry forest. Ethnobiology and Conservation. 2022;11.
113. Du Z, He Y, Wang H, Wang C, Duan Y. Potential geographical distribution and habitat shift of the genus Ammopiptanthus in China under current and future climate change based on the MaxEnt model. Journal of Arid Environments. 2021;184:104328.
114. Zhang F-G, Liang F, Wu K, Xie L, Zhao G, Wang Y. The potential habitat of Angelica dahurica in China under climate change scenario predicted by Maxent model. Frontiers in plant science. 2024;15:1388099.
115. Chen C, Wang B, Li J, Xiao Y, Chen K, Liu N, et al. Predicting potential and quality distribution of Anisodus tanguticus (Maxim.) Pascher under different climatic conditions in the Qinghai–Tibet plateau. Frontiers in Plant Science. 2024;15:1369641.
116. Bhat IA, Fayaz M, Rafiq S, Guleria K, Qadir J, Wani TA, et al. Predicting potential distribution and range dynamics of Aquilegia fragrans under climate change: Insights from ensemble species distribution modelling. Environmental Monitoring and Assessment. 2023;195(5):623.
117. Xu J, Wu Y, Wang S, Xu Y, Du C, Rohani ER, et al. Predicting the potential distribution of Arisaema heterophyllum in China under current and future climate change based on ArcGIS and MaxEnt model. Plant Biosystems-An International Journal Dealing with all Aspects of Plant Biology. 2024;158(6):1326-34.

118. Wani ZA, Pant S, Bhat JA, Shukla G. Distribution and survival of medicinal and aromatic plants is threatened by the anticipated climate change. Trees, Forests and People. 2024;16:100549.
119. Sarma B, Baruah PS, Tanti B. Habitat distribution modeling for reintroduction and conservation of Aristolochia indica L.-a threatened medicinal plant in Assam, India. Journal of Threatened Taxa. 2018;10(11):12531-7.
120. Wang D, Shi C, Alamgir K, Kwon S, Pan L, Zhu Y, et al. Global assessment of the distribution and conservation status of a key medicinal plant (Artemisia annua L.): The roles of climate and anthropogenic activities. Science of the Total Environment. 2022;821:153378.
121. Zhang T, Lv X, Zhao Q, Zhang C, Yin H, Deng S, et al. Assessment of suitable region of Asparagus cochinchinensis (Lour.) Merr. under different climatic conditions in China by the MaxEnt model and HPLC analysis. Ecology and Evolution. 2024;14(10):e70354.
122. Guo J, Zhang C, Zhang M, Bi Y, Wang M, Li M. Analysis of the distribution of Astragalus membranaceus var. Mongholicus in Inner Mongolia under climate change using the GEE platform. Science of Traditional Chinese Medicine. 2024;2(3):237-44.
123. Dong P, Wang L, Qiu D, Liang W, Cheng J, Wang H, et al. Evaluation of the environmental factors influencing the quality of Astragalus membranaceus var. mongholicus based on HPLC and the Maxent model. BMC Plant Biology. 2024;24(1):697.
124. Guan L, Yang Y, Jiang P, Mou Q, Gou Y, Zhu X, et al. Potential distribution of Blumea balsamifera in China using MaxEnt and the ex situ conservation based on its effective components and fresh leaf yield. Environmental Science and Pollution Research. 2022;29(29):44003-19.
125. Kolanowska M. Climate change will decrease the coverage of suitable niches for Asian medicinal orchid (Bulbophyllum odoratissimum) and its main phorophyte (Pistacia weinmannifolia). Scientific Reports. 2024;14(1):22656.
126. Wouyou HG, Lokonon BE, Idohou R, Zossou-Akete AG, Assogbadjo AE, Kakaï RG. Predicting the potential impacts of climate change on the endangered Caesalpinia bonduc (L.) Roxb in Benin (West Africa). Heliyon. 2022;8(3).
127. Ashraf U, Chaudhry MN, Ahmad SR, Ashraf I, Arslan M, Noor H, et al. Impacts of climate change on Capparis spinosa L. based on ecological niche modeling. PeerJ. 2018;6:e5792.
128. de Albergaria ET, de Oliveira AFM, Albuquerque UP. Effect of rainfall and soil fertility on total phenol and tannin contents in Cenostigma microphyllum (Mart. ex G. Don) E. Gagnon & GP Lewis (Fabaceae). Acta Physiologiae Plantarum. 2021;43:1-8.
129. Xie C, Huang B, Jim CY, Liu D, Liu C, Zhu Z. Predicting suitable habitat for the endangered plant Cephalotaxus oliveri Mast. in China. Environmental Conservation. 2023;50(1):50-7.
130. Su Q, Du Z, Xue Y, Li H, Zhang Y, Zhang S, et al. Habitat Suitability Modeling of Endemic Genus Chimonanthus in China under Climate Change. Forests (19994907). 2024;15(9).
131. Qi S, Luo W, Chen K-L, Li X, Luo H-L, Yang Z-Q, et al. The prediction of the potentially suitable distribution area of Cinnamomum mairei H. Lév in China based on the MaxEnt model. Sustainability. 2022;14(13):7682.
132. Fang HQ, Jiang ZX, Chen SM, Xie T, Xue Y, Song J, et al. Predicting the distribution of potentially suitable habitat in China for Cirsium japonicum Fisch. ex DC. under future climate scenarios based on the R-optimized MaxEnt model. Ecology and Evolution. 2024;14(7):e11653.
133. Shao M, Wang L, Li B, Li S, Fan J, Li C. Maxent modeling for identifying the nature reserve of cistanche deserticola ma under effects of the host (Haloxylon Bunge) forest and climate changes in Xinjiang, China. Forests. 2022;13(2):189.

134. Xia Y, Kazim M, Nabeel Nasir M, Yang Y, Li Q, Li T, et al. Suitability changes of Citrus medica L. var. sarcodactylis Swingle, a medicine-food plants affected by climate warming using the optimized MaxEnt model. PLoS One. 2023;18(3):e0282659.
135. Purohit S, Rawat N. MaxEnt modeling to predict the current and future distribution of Clerodendrum infortunatum L. under climate change scenarios in Dehradun district, India. Modeling Earth Systems and Environment. 2022;8(2):2051-63.
136. Lu Z, Shan Y, Shan H, Wen H, Wu Y, Han R, et al. Effect of climate change on Clinopodium polycephalum (Vaniot) CY Wu & SJ Hsuan distribution adopting temporal data, ArcGIS, and the MaxEnt model. Frontiers in Plant Science. 2024;15:1445764.
137. Lee H, Koo HJ, Lee KC, Song Y, Joo W-K, Chae C-J. Prediction and Classification of Phenol Contents in Cnidium officinale Makino Using a Stacking Ensemble Model in Climate Change Scenarios. Agronomy. 2024;14(8):1766.
138. Li J, Fan G, He Y. Predicting the current and future distribution of three Coptis herbs in China under climate change conditions, using the MaxEnt model and chemical analysis. Science of the Total Environment. 2020;698:134141.
139. Radha KO, Khwarahm NR. An integrated approach to map the impact of climate change on the distributions of Crataegus azarolus and Crataegus monogyna in Kurdistan Region, Iraq. Sustainability. 2022;14(21):14621.
140. Li W-N, Zhao Q, Guo M-H, Lu C, Huang F, Wang Z-Z, et al. Predicting the potential distribution of the endangered plant Cremastra appendiculata (Orchidaceae) in China under multiple climate change scenarios. Forests. 2022;13(9):1504.
141. Boral D, Moktan S. Modelling current and future potential distribution of medicinal orchids in Darjeeling eastern Himalaya. Plant Ecology. 2024;225(3):213-26.
142. Pourmeidani A, Ghamghami M, Olya H, Ghahreman N. Determination of suitable regions for cultivation of three medicinal plants under a changing climate. Environmental Processes. 2020;7:89-108.
143. Wang P, Jin Z, Yu T, Qin K-q, Sang X-y, Tao J-p, et al. Prediction of the potential distribution of Curcuma in China under current and future climate scenarios. Acta Prataculturae Sinica. 2024;33(10):14.
144. Xu Y, Huang Y, Zhao H, Yang M, Zhuang Y, Ye X. Modelling the effects of climate change on the distribution of endangered Cypripedium japonicum in China. Forests 12: 429. 2021.
145. Kunwar RM, Rimal B, Sharma HP, Poudel RC, Pyakurel D, Tiwari A, et al. Distribution and habitat modeling of Dactylorhiza hatagirea (D. Don) Soo, Paris polyphylla Sm. and Taxus species in Nepal Himalaya. Journal of Applied Research on Medicinal and Aromatic Plants. 2021;20:100274.
146. Tarnian F, Kumar S, Azarnivand H, Chahouki MAZ, Mossivand AM. Assessing the effects of climate change on the distribution of Daphne mucronata in Iran. Environmental Monitoring and Assessment. 2021;193(9):562.
147. Abolmaali SM-R, Tarkesh M, Bashari H. MaxEnt modeling for predicting suitable habitats and identifying the effects of climate change on a threatened species, Daphne mucronata, in central Iran. Ecological Informatics. 2018;43:116-23.
148. Zuo J, Tang X, Zhang H, Zu M, Zhang X, Yuan Y. Analysis of niche shift and potential suitable distributions of Dendrobium under the impact of global climate change. Environmental Science and Pollution Research. 2023;30(5):11978-93.
149. Tang X, Yuan Y, Zhang J. How climate change will alter the distribution of suitable Dendrobium habitats. Frontiers in Ecology and Evolution. 2020;8:536339.

150. Tafesse B, Bekele T, Demissew S, Dullo BW, Nemomissa S, Chala D. Conservation implications of mapping the potential distribution of an Ethiopian endemic versatile medicinal plant, Echinops kebericho Mesfin. Ecology and Evolution. 2023;13(5):e10061.
151. Li Y, Wang Y, Zhao C, Du X, He P, Meng F. Predicting the spatial distribution of three Ephedra species under climate change using the MaxEnt model. Heliyon. 2024;10(12).
152. Anand A, Garg VK. Modeling the species occurrence probability and response of climate change on Himalayan Somalata plant under different shared socioeconomic pathways. Environmental Monitoring and Assessment. 2024;196(7):647.
153. Li X, Gu X, Mao F, Guo H, Qiu J, Liu Y, et al. Assessment of the potential habitat suitability and ephedrine quality of two Ephedra species in China under climate change. Plant Biosystems-An International Journal Dealing with all Aspects of Plant Biology. 2024;158(3):479-89.
154. Zhang K, Liu Z, Abdukeyum N, Ling Y. Potential geographical distribution of medicinal plant ephedra sinica stapf under climate change. Forests. 2022;13(12):2149.
155. Xie YF, Yang L, Deng RY, Chen MH, Luan XF, Gottardi E, et al. Changes in the range of the medicinal herb Eriocaulon buergerianum Körnicke.(Eriocaulaceae) under climate change. Plant Biology. 2018;20(4):771-9.
156. Xie S, Si H, Sun H, Zhao Q, Li X, Wang S, et al. Predicting the potential distribution of the endangered plant Eucommia ulmoides in China under the background of climate change, Sustainability. Sustainability. 2023;54.
157. Zhang K, Sun L, Tao J. Impact of climate change on the distribution of Euscaphis japonica (Staphyleaceae) trees. Forests 11: 525. 2020.
158. Wang E, Lu Z, Rohani ER, Ou J, Tong X, Han R. Current and future distribution of Forsythia suspensa in China under climate change adopting the MaxEnt model. Frontiers in Plant Science. 2024;15:1394799.
159. Rana SK, Rana HK, Ghimire SK, Shrestha KK, Ranjitkar S. Predicting the impact of climate change on the distribution of two threatened Himalayan medicinal plants of Liliaceae in Nepal. Journal of Mountain Science. 2017;14:558-70.
160. Liu L, Zhang Y, Huang Y, Zhang J, Mou Q, Qiu J, et al. Simulation of potential suitable distribution of original species of Fritillariae Cirrhosae Bulbus in China under climate change scenarios. Environmental Science and Pollution Research. 2022:1-14.
161. Naghipour Borj AA, Ostovar Z, Asadi E. The influence of climate change on distribution of an endangered medicinal plant (Fritillaria Imperialis L.) in central Zagros. Journal of Rangeland Science. 2019;9(2):159-71.
162. Pramanik M, Paudel U, Mondal B, Chakraborti S, Deb P. Predicting climate change impacts on the distribution of the threatened Garcinia indica in the Western Ghats, India. Climate Risk Management. 2018;19:94-105.
163. Hu J, Feng Y, Zhong H, Liu W, Tian X, Wang Y, et al. Impact of climate change on the geographical distribution and niche dynamics of Gastrodia elata. PeerJ. 2023;11:e15741.
164. Yan H, He J, Zhao Y, Zhang L, Zhu C, Wu D. Gentiana macrophylla response to climate change and vulnerability evaluation in China. Global Ecology and Conservation. 2020;22:e00948.
165. Zou H, Chen B, Zhang B, Zhou X, Zhang X, Zhang X, et al. Conservation planning for the endemic and endangered medicinal plants under the climate change and human disturbance: a case study of Gentiana manshurica in China. Frontiers in Plant Science. 2023;14:1184556.
166. Zhang H, Sun X, Zhang G, Zhang X, Miao Y, Zhang M, et al. Potential global distribution of the habitat of endangered Gentiana rhodantha Franch: Predictions based on MaxEnt ecological niche modeling. Sustainability. 2022;15(1):631.

167. Zhang J, Zhang Z, Wang Y, Zuo Y, Cai C. Environmental impact on the variability in quality of Gentiana rigescens, a medicinal plant in southwest China. Global ecology and conservation. 2020;24:e01374.
168. Bidak LM, Heneidy SZ, Halmy MWA, El-Kenany ET. Sustainability potential for Ginkgo biloba L. plantations under climate change uncertainty: An ex-situ conservation perspective. Acta Ecologica Sinica. 2022;42(2):101-14.
169. Guo Y, Wang M, Gao C, Fu F-F, El-Kassaby YA, Wang T, et al. Spatial prediction and delineation of Ginkgo biloba production areas under current and future climatic conditions. Industrial Crops and Products. 2021;166:113444.
170. Huang Z, Xie L, Wang H, Zhong J, Li Y, Liu J, et al. Geographic distribution and impacts of climate change on the suitable habitats of Zingiber species in China. Industrial Crops and Products. 2019;138:111429.
171. Li M, Zhang Y, Yang Y, Wang T, Wu C, Zhang X. Prediction of Historical, Current, and Future Configuration of Tibetan Medicinal Herb Gymnadenia orchidis Based on the Optimized MaxEnt in the Qinghai–Tibet Plateau. Plants. 2024;13(5):645.
172. Fassou G, Kougioumoutzis K, Iatrou G, Trigas P, Papasotiropoulos V. Genetic diversity and range dynamics of Helleborus odorus subsp. cyclophyllus under different climate change scenarios. Forests. 2020;11(6):620.
173. Yi Y-j, Cheng X, Yang Z-F, Zhang S-H. Maxent modeling for predicting the potential distribution of endangered medicinal plant (H. riparia Lour) in Yunnan, China. Ecological Engineering. 2016;92:260-9.
174. Yi Y-j, Zhou Y, Cai Y-p, Yang W, Li Z-w, Zhao X. The influence of climate change on an endangered riparian plant species: The root of riparian Homonoia. Ecological Indicators. 2018;92:40-50.
175. Liu L, Guan L, Zhao H, Huang Y, Mou Q, Liu K, et al. Modeling habitat suitability of Houttuynia cordata Thunb (Ceercao) using MaxEnt under climate change in China. Ecological Informatics. 2021;63:101324.
176. Cao Z, Zhang L, Zhang X, Guo Z. Predicting the potential distribution of Hylomecon japonica in China under current and future climate change based on Maxent model. Sustainability. 2021;13(20):11253.
177. Guidigan MLG, Azihou F, Idohou R, Okhimamhe AA, Fandohan AB, Sinsin B, et al. Modelling the current and future distribution of Kigelia africana under climate change in Benin, West Africa. Modeling Earth Systems and Environment. 2018;4:1225-38.
178. Akyol A, Örücü ÖK, Arslan ES, Sarıkaya AG. Predicting of the current and future geographical distribution of Laurus nobilis L. under the effects of climate change. Environmental Monitoring and Assessment. 2023;195(4):459.
179. wa Rusaati BI, Kang JW. MaxEnt modeling for predicting the potential distribution of Lebrunia bushaie Staner (Clusiaceae) under different climate change scenarios in Democratic Republic of Congo. Journal of Asia-Pacific Biodiversity. 2024;17(1):1-6.
180. Chen C, Zhou L, Han Y, Wen J, Liu L, Liu Q, et al. Comprehensive analysis of the effects of climate change on the species distribution and active components of Leonurus japonicus Houtt. Industrial Crops and Products. 2024;218:119017.
181. Wang Y, Xie L, Zhou X, Chen R, Zhao G, Zhang F. Prediction of the potentially suitable areas of Leonurus japonicus in China based on future climate change using the optimized MaxEnt model. Ecology and Evolution. 2023;13(10):e10597.

182. Liu L, Shi B, Li J, Wen J, Zhou L, He Y. Assessing environmental suitability of Ligusticum chuanxiong based on ecological analyses with chemical and molecular verification. Heliyon. 2023;9(3).
183. Dhyani A, Kadaverugu R, Nautiyal BP, Nautiyal MC. Predicting the potential distribution of a critically endangered medicinal plant Lilium polyphyllum in Indian Western Himalayan Region. Regional Environmental Change. 2021;21(2):30.
184. Bai J, Wang H, Hu Y. Prediction of Potential Suitable Distribution of Liriodendron chinense (Hemsl.) Sarg. in China Based on Future Climate Change Using the Optimized MaxEnt Model. Forests. 2024;15(6):988.
185. Gupta R, Sharma LK, Rajkumar M, Mohammad N, Khan ML. Predicting habitat suitability of Litsea glutinosa: a declining tree species, under the current and future climate change scenarios in India. Landscape and Ecological Engineering. 2023;19(2):211-25.
186. Wu X, Song L, Yang Q, Yang Y, Hu M. Assessing the impact of climate change on the habitat dynamics of Magnolia biondii in China: A Maxent modelling approach. . Applied Ecology & Environmental Research. 2024;22(3).
187. Song C, Liu H, Gao J. Habitat preference and potential distribution of Magnolia officinalis subsp. officinalis and M. o. subsp. biloba in China. Nature Conservation. 2019;36:93-111.
188. Paul S, Samant S. Population ecology and habitat suitability modelling of an endangered and endemic medicinal plant Meconopsis aculeata Royle under projected climate change in the Himalaya. Environmental and Experimental Botany. 2024;225:105837.
189. Shi N, Naudiyal N, Wang J, Gaire NP, Wu Y, Wei Y, et al. Assessing the impact of climate change on potential distribution of Meconopsis punicea and its influence on ecosystem services supply in the southeastern margin of Qinghai-Tibet Plateau. Frontiers in Plant Science. 2022;12:830119.
190. Soilhi Z, Sayari N, Benalouache N, Mekki M. Predicting current and future distributions of Mentha pulegium L. in Tunisia under climate change conditions, using the MaxEnt model. Ecological Informatics. 2022;68:101533.
191. Ali F, Khan N, Khan AM, Ali K, Abbas F. Species distribution modelling of Monotheca buxifolia (Falc.) A. DC.: Present distribution and impacts of potential climate change. Heliyon. 2023;9(2).
192. Liang J, Tang G, Qin X. Delineating the Area for Sustainable Cultivation of Morinda officinalis Based on the MaxEnt Model. Agronomy. 2024;14(6):1134.
193. Bania JK, Deka JR, Hazarika A, Das AK, Nath AJ, Sileshi GW. Modelling habitat suitability for Moringa oleifera and Moringa stenopetala under current and future climate change scenarios. Scientific Reports. 2023;13(1):20221.
194. Borges LL, Alves SF, Sampaio BL, Conceição EC, Bara MTF, Paula JR. Environmental factors affecting the concentration of phenolic compounds in Myrcia tomentosa leaves. Revista Brasileira de Farmacognosia. 2013;23(2):230-8.
195. Remya K, Ramachandran A, Jayakumar. Predicting the current and future suitable habitat distribution of Myristica dactyloides Gaertn. using MaxEnt model in the Eastern Ghats, India. Ecological engineering. 2015;82:184-8.
196. Li J, Wu J, Peng K, Fan G, Yu H, Wang W, et al. Simulating the effects of climate change across the geographical distribution of two medicinal plants in the genus Nardostachys. PeerJ. 2019;7:e6730.

197. Wen J, Zhou L, Liu L, He Y. Analysis of the impact of climate change on the distribution and active compound content of the plateau medicinal plant Nardostachys jatamansi (D. Don) DC. Industrial Crops and Products. 2022;187:115438.
198. Karami S, Ejtehadi H, Moazzeni H, Vaezi J, Behroozian M. Minimal climate change impacts on the geographic distribution of Nepeta glomerulosa, medicinal species endemic to southwestern and central Asia. Scientific Reports. 2022;12(1):19893.
199. Zhao Z, Guo Y, Wei H, Ran Q, Liu J, Zhang Q, et al. Potential distribution of Notopterygium incisum Ting ex HT Chang and its predicted responses to climate change based on a comprehensive habitat suitability model. Ecology and Evolution. 2020;10(6):3004-16.
200. Kumar D, Rawat S, Joshi R. Predicting the current and future suitable habitat distribution of the medicinal tree Oroxylum indicum (L.) Kurz in India. Journal of Applied Research on Medicinal and Aromatic Plants. 2021;23:100309.
201. Kong F, Tang L, He H, Yang F, Tao J, Wang W. Assessing the impact of climate change on the distribution of Osmanthus fragrans using Maxent. Environmental Science and Pollution Research. 2021;28:34655-63.
202. Huang X-M, Zhao C, Cai K, Huang Y. Climatic Changes in the Anthropocene Have Increased the Suitable Habitat Areas of Paeonia delavayi in China. Polish Journal of Environmental Studies. 2023;32(5).
203. Peng L-P, Cheng F-Y, Hu X-G, Mao J-F, Xu X-X, Zhong Y, et al. Modelling environmentally suitable areas for the potential introduction and cultivation of the emerging oil crop Paeonia ostii in China. Scientific Reports. 2019;9(1):3213.
204. Zhang K, Zhang Y, Tao J. Predicting the potential distribution of Paeonia veitchii (Paeoniaceae) in China by incorporating climate change into a maxent model. Forests. 2019;10(2):190.
205. Du Z, Wu J, Meng X, Li J, Huang L. Predicting the Global Potential Distribution of Four Endangered Panax Species in Middle-and Low-Latitude Regions of China by the Geographic Information System for Global Medicinal Plants (GMPGIS). Molecules.22.
206. Liang S, Xi-Wen L, Xiang-Xiao M, Jie W, Huan T, Shui-Ming X, et al. Prediction of the globally ecological suitability of Panax quinquefolius by the geographic information system for global medicinal plants (GMPGIS). Chinese Journal of Natural Medicines. 2019;17(7):481-9.
207. Hong-Qun L, Xiao-Long P, Peng J, Xie-Ping S, Ligang X. Geospatial Distribution Variation of the Paris Polyphylla (Melanthiaceae) In China Under Climate Change Scenario. Pak J Bot. 2024;56(3):1067-74.
208. Bao R, Li X, Zheng J. Feature tuning improves MAXENT predictions of the potential distribution of Pedicularis longiflora Rudolph and its variant. PeerJ 10: e13337. 2022.
209. Chen T, Acma F, Amoroso V, Medecilo Guiang M, Huang B. Distribution of climatic suitability of Pellionia scabra Benth.(Urticaceae) in China. Applied Ecology & Environmental Research. 2022;20(5).
210. Khanum R, Mumtaz A, Kumar S. Predicting impacts of climate change on medicinal asclepiads of Pakistan using Maxent modeling. Acta Oecologica. 2013;49:23-31.
211. Zhang B, Zou H, Chen B, Zhang X, Kang X, Wang C, et al. Optimizing the distribution pattern of species under climate change: The protection and management of Phellodendron amurense in China. Frontiers in Ecology and Evolution. 2023;11:1186627.
212. Kumar R, Joshi R, Kumari M, Thakur R, Kumar D, Kumar S. Elevated CO2 and temperature influence key proteins and metabolites associated with photosynthesis, antioxidant and carbon metabolism in Picrorhiza kurroa. Journal of proteomics. 2020;219:103755.

213. Rawat N, Purohit S, Painuly V, Negi GS, Bisht MPS. Habitat distribution modeling of endangered medicinal plant Picrorhiza kurroa (Royle ex Benth) under climate change scenarios in Uttarakhand Himalaya, India. Ecological Informatics. 2022;68:101550.
214. Monteiro WP, de Souza EB, Miranda LdS, Anjos LJ, Caldeira CF. Potential Distribution of Pilocarpus microphyllus in the Amazonia/Cerrado Biomes under Near-Future Climate Change Scenarios. Plants. 2023;12(11):2106.
215. Qiu J, Gu X, Li X, Bi J, Liu Y, Zheng K, et al. Identification of potentially suitable areas for nucleosides of Pinellia Ternata (Thunb.) Breit using ecological niche modeling. Environmental Monitoring and Assessment. 2023;195(12):1479.
216. HamadAmin BA, Khwarahm NR. Mapping impacts of climate change on the distributions of two endemic tree species under socioeconomic pathway scenarios (SSP). Sustainability. 2023;15(6):5469.
217. Jiang H, Liu T, Li L, Zhao Y, Pei L, Zhao J. Predicting the potential distribution of Polygala tenuifolia Willd. under climate change in China. PLoS One. 2016;11(9):e0163718.
218. Guo Y, Zhang S, Tang S, Pan J, Ren L, Tian X, et al. Analysis of the prediction of the suitable distribution of Polygonatum kingianum under different climatic conditions based on the MaxEnt model. Frontiers in Earth Science. 2023;11:1111878.
219. Chen K, Wang B, Chen C, Zhou G. MaxEnt modeling to predict the current and future distribution of Pomatosace filicula under climate change scenarios on the Qinghai–Tibet Plateau. Plants. 2022;11(5):670.
220. Li Z, Rubert-Nason KF, Jamieson MA, Raffa KF, Lindroth RL. Root secondary metabolites in Populus tremuloides: effects of simulated climate warming, defoliation, and genotype. Journal of chemical ecology. 2021;47(3):313-21.
221. Tadesse Z, Nemomissa S, Lemessa D. Predicting the distributions of Pouteria adolfi-friederici and Prunus africana tree species under current and future climate change scenarios in Ethiopia. African Journal of Ecology. 2023;61(1):204-16.
222. Mbatudde M, Mwanjololo M, Kakudidi EK, Dalitz H. Modelling the potential distribution of endangered P runus africana (H ook. f.) K alkm. in E ast A frica. African Journal of Ecology. 2012;50(4):393-403.
223. Khanal S, Timilsina R, Behroozian M, Peterson AT, Poudel M, Alwar MSS, et al. Potential impact of climate change on the distribution and conservation status of Pterocarpus marsupium, a Near Threatened South Asian medicinal tree species. Ecological Informatics. 2022;70:101722.
224. Xu W, Zhu S, Yang T, Cheng J, Jin J. Maximum entropy niche-based modeling for predicting the potential suitable habitats of a traditional medicinal plant (Rheum nanum) in Asia under climate change conditions. Agriculture. 2022;12(5):610.
225. Wani IA, Verma S, Kumari P, Charles B, Hashim MJ, El-Serehy HA. Ecological assessment and environmental niche modelling of Himalayan rhubarb (Rheum webbianum Royle) in northwest Himalaya. PLoS One. 2021;16(11):e0259345.
226. Yang M, Sun L, Yu Y, Zhang H, Malik I, Wistuba M, et al. Predicting the potential geographical distribution of Rhodiola L. in China under climate change scenarios. Plants. 2023;12(21):3735.
227. Xie C, Huang B, Jim CY, Han W, Liu D. Predicting differential habitat suitability of Rhodomyrtus tomentosa under current and future climate scenarios in China. Forest Ecology and Management. 2021;501:119696.

228. Wang Y, Liu H, Xu J, Yu S, Huang Y, Zhang Y, et al. Prediction of suitable planting areas of Rubia cordifolia in China based on a species distribution model and analysis of specific secondary metabolites. Industrial Crops and Products. 2023;206:117651.
229. Karalija E, Dahija S, Tarkowski P, Zeljković SĆ. Influence of climate-related environmental stresses on economically important essential oils of Mediterranean Salvia sp. Frontiers in Plant Science. 2022;13:864807.
230. Ardestani EG, Ghahfarrokhi ZH. Ensembpecies distribution modeling of Salvia hydrangea under future climate change scenarios in Central Zagros Mountains, Iran. Global Ecology and Conservation. 2021;26:e01488.
231. Luo W, Han S, Yu T, Wang P, Ma Y, Wan M, et al. Assessing the suitability and dynamics of three medicinal Sambucus species in China under current and future climate scenarios. Frontiers in Plant Science. 2023;14:1194444.
232. Liao J, Yang C, Shao Q, Sun Q, Han Y. Construction of an ecological model of Sambucus javanica blume in China under different climate scenarios based on maxent model. Plant Ecology. 2023;224(3):221-37.
233. Chen B, Zou H, Zhang B, Zhang X, Jin X, Wang C, et al. Distribution pattern and change prediction of Saposhnikovia divaricata suitable area in China under climate change. Ecological Indicators. 2022;143:109311.
234. Jena M, Mohanta MR, Charles B, Aravind N, Ravikanth G, Sahu SC. Predicting potential distribution, range change and niche dynamics for Saraca asoca (Roxb.) De Wilde: a threatened medicinal plant under climatic change. Current Science. 2023;125(9):989.
235. Ouyang X, Bai S, Strachan GB, Chen A. Simulation of the potential distribution of rare and endangered Satyrium species in China under climate change. Ecology and Evolution. 2022;12(7):e9054.
236. Shang J, Zhao Q, Yan P, Sun M, Sun H, Liang H, et al. Environmental factors influencing potential distribution of Schisandra sphenanthera and its accumulation of medicinal components. Frontiers in Plant Science. 2023;14:1302417.
237. Rong W, Huang X, Hu S, Zhang X, Jiang P, Niu P, et al. Impacts of climate change on the habitat suitability and natural product accumulation of the medicinal plant Sophora alopecuroides L. based on the MaxEnt Model. Plants. 2024;13(11):1424.
238. Shaban M, Ghehsareh Ardestani E, Ebrahimi A, Borhani M. Climate change impacts on optimal habitat of Stachys inflata medicinal plant in central Iran. Scientific Reports. 2023;13(1):6580.
239. Tomaz JS, Bezerra CdS, Aguiar AVd, Wrege MS, Lopes MTG. Prediction of the natural distribution, habitat and conservation of Stryphnodendron pulcherrimum (Willd.) Hochr. in response to global climate change. Pesquisa Agropecuária Tropical. 2022;52:e72422.
240. Saputra MH, Lee HS. Evaluation of climate change impacts on the potential distribution of Styrax sumatrana in North Sumatra, Indonesia. Sustainability. 2021;13(2):462.
241. Wani BA, Wani SA, Magray JA, Ahmad R, Ganie AH, Nawchoo IA. Habitat suitability, range dynamics, and threat assessment of Swertia petiolata D. Don: a Himalayan endemic medicinally important plant under climate change. Environmental Monitoring and Assessment. 2023;195(1):214.
242. Kurpis J, Serrato-Cruz MA, Arroyo TPF. Modeling the effects of climate change on the distribution of Tagetes lucida Cav.(Asteraceae). Global Ecology and Conservation. 2019;20:e00747.

243. Nimasow G, Dai Nimasow O, Tsering G. Vanishing Taxus baccata L. due to unsustainable exploitation and climate change in west Kameng and Tawang districts of Arunachal Pradesh. Earth. 2015;4(3-1):11-8.
244. Rathore P, Roy A, Karnatak H. Modelling the vulnerability of Taxus wallichiana to climate change scenarios in South East Asia. Ecological Indicators. 2019;102:199-207.
245. Arshad F, Waheed M, Fatima K, Harun N, Iqbal M, Fatima K, et al. Predicting the suitable current and future potential distribution of the native endangered tree Tecomella undulata (Sm.) Seem. in Pakistan. Sustainability. 2022;14(12):7215.
246. Kailash B, Charles B, Ravikanth G, Setty S, Kadirvelu K. Identifying the potential global distribution and conservation areas for Terminalia chebula, an important medicinal tree species under changing climate scenario. Tropical Ecology. 2022;63(4):584-95.
247. Wang J, Qian Q, Zhang F, Jia X, He J. The possible future changes in potential suitable habitats of Tetrastigma hemsleyanum (Vitaceae) in China predicted by an ensemble model. Global Ecology and Conservation. 2022;35:e02083.
248. Hosseini N, Ghorbanpour M, Mostafavi H. The influence of climate change on the future distribution of two Thymus species in Iran: MaxEnt model-based prediction. BMC Plant Biology. 2024;24(1):269.
249. Hosseini N, Mostafavi H, Ghorbanpour M. The future range of two Thymus daenensis subspecies in Iran under climate change scenarios: MaxEnt model-based prediction. Genetic Resources and Crop Evolution. 2025;72(1):717-34.
250. Hosseini N, Ghorbanpour M, Mostafavi H. Habitat potential modelling and the effect of climate change on the current and future distribution of three Thymus species in Iran using MaxEnt. Scientific Reports. 2024;14(1):3641.
251. Laftouhi A, Slimani M, Elrherabi A, Bouhrim M, Mahraz MA, Idrissi AM, et al. Effect of Temperature and Water Stress on the Antioxidant and Antidiabetic Activities of Thymus vulgaris Essential Oil. Tropical Journal of Natural Product Research. 2024;8(1).
252. Zhang F-G, Zhang S, Wu K, Zhao R, Zhao G, Wang Y. Potential habitat areas and priority protected areas of Tilia amurensis Rupr in China under the context of climate change. Frontiers in Plant Science. 2024;15:1365264.
253. Zhang H, Li Z, Zou H, Wang Z, Zhu X, Zhang Y, et al. Global Warming Drives Transitions in Suitable Habitats and Ecological Services of Rare Tinospora Miers Species in China. Diversity. 2024;16(3):181.
254. Nagahama N, Bonino MF. Modeling the potential distribution of Valeriana carnosa Sm. in Argentinean Patagonia: A proposal for conservation and in situ cultivation considering climate change projections. Journal of Applied Research on Medicinal and Aromatic Plants. 2020;16:100240.
255. Kumari P, Wani IA, Khan S, Verma S, Mushtaq S, Gulnaz A, et al. Modeling of Valeriana wallichii habitat suitability and niche dynamics in the Himalayan Region under anticipated climate change. Biology. 2022;11(4):498.
256. Senkoro AM, Munt DD, Shackleton CM, Ribeiro-Barros AI, Voeks RA. The case of a threatened medicinal tree with optimistic prospects under climate change. Global Ecology and Conservation. 2024;54:e03126.
257. Tian P, Liu Y, Sui M, Ou J. Prediction of potential habitats of Zanthoxylum armatum DC. and their changes under climate change. Sustainability. 2022;14(19):12422.

258. Yang Y, He J, Liu Y, Zeng J, Zeng L, He R, et al. Assessment of Chinese suitable habitats of Zanthoxylum nitidum in different climatic conditions by Maxent model, HPLC, and chemometric methods. Industrial Crops and Products. 2023;196:116515.
259. Huang B, Chen S, Xu L, Jiang H, Chen X, He H, et al. Predicting the potential geographical distribution of Zingiber striolatum Diels (Zingiberaceae), a medicine food homology plant in China. Scientific Reports. 2024;14(1):22206.
260. Zhao Q, Mi ZY, Lu C, Zhang XF, Chen LJ, Wang SQ, et al. Predicting potential distribution of Ziziphus spinosa (Bunge) HH Hu ex FH Chen in China under climate change scenarios. Ecology and Evolution. 2022;12(2):e8629.
